# Intrinsic resistance to short-tailed azoles in the basal fungus *Mucor lusitanicus*: functional analysis of Cyp51 isoforms and amino acid substitutions

**DOI:** 10.3389/fmicb.2025.1702408

**Published:** 2025-11-26

**Authors:** K. Rosam, M. V. Keniya, L.-M. Zenz, C. Müller, B. Sarg, M. Stojanovic, U. Binder, B. C. Monk, M. Lackner

**Affiliations:** 1 Institute for Hygiene and Medical Microbiology, Medical University Innsbruck, Innsbruck, Austria; 2 Department of Oral Sciences, Sir John Walsh Research Institute, University of Otago, Dunedin, New Zealand; 3 Hackensack Meridian Health Center for Discovery and Innovation, Hackensack, NJ, United States; 4 Department of Pharmacy, Center for Drug Research, Ludwig-Maximilians-Universität München, Munich, Germany; 5 Institute of Medical Biochemistry, Protein Core Facility, Medical University Innsbruck, Innsbruck, Austria

**Keywords:** azole, AMR (antimicrobial resistance), mucormycoses, mucormycetes, lanosterol 14α demethylase, *Saccharomyces cerevisiae*, heterologous expression, GMO (genetically modified organism)

## Abstract

**Introduction:**

The basal fungus *Mucor lusitanicus* (*Mlu*) is a cause of mucormycosis, with limited treatment options due to intrinsic resistance to short-tailed azoles (fluconazole, voriconazole) and echinocandins. *In silico* analysis identified amino-acid-substitutions Y129F and V293A in the substrate-binding pocket of sterol-14α-demethylase (Cyp51, Erg11) isoform F5 as potential resistance mechanisms.

**Methods:**

This hypothesis has been experimentally tested for *M. lusitanicus* by expressing its Cyp51 isoforms (MluCyp51-F1, MluCyp51-F5) and modified MluCyp51-F5 variants (F129Y, A293V, F129Y A293V) with/without their cognate NADPH-cytochrome-P450-reductase (MluCPR) in *Saccharomyces cerevisiae*.

**Results:**

Strains expressing MluCyp51 isoforms +/− MluCPR showed expression levels of 38–69% compared to an overexpressed Erg11 control. Susceptibility assays confirmed that MluCyp51-F5 confers intrinsic resistance to short-tailed azoles, while substitutions F129Y, A293V, or both restored susceptibility. Growth- and susceptibility-assays revealed that the MluCyp51-F1 + CPR construct had a voriconazole MIC of 0.5 μM, while MluCyp51-F5 + CPR had a MIC of 11.5 μM, with no changes in growth rate or ergosterol composition at 1.0 μM voriconazole. Susceptibility to long-tailed azoles (e.g., posaconazole) remained unchanged for both isoforms.

**Discussion:**

These findings demonstrate the functional expression of MluCyp51-F1 and MluCyp51-F5 isoforms in a phylogenetically distant host and confirm that conserved substitutions Y129F and V293A in MluCyp51-F5 confer intrinsic resistance to short-tailed azoles in *M. lusitanicus*.

## Introduction

Mucorales are basal fungi in the Mucoromycota phylum, with an estimated divergence time of approximately 581 million years ago (Zhao et al., 2023). In the last four decades, their position within the phylogenetic tree has undergone significant revision (Voigt et al., 2021; Liu et al., 2006; James et al., 2006). Currently, the Mucoromycota phylum is classified into three subdivisions and is recognized as a sister clade to Dikarya (Walther et al., 2019). The Mucorales order includes *Mucor lusitanicus*, a common cause of mucormycosis in Europe (Skiada et al., 2018; Cornely et al., 2019). This rapidly progressing and highly destructive infection is associated with poor outcomes and mortality rates ranging from 30–80% for disseminated infections (Cornely et al., 2019; Roden et al., 2005). The incidence of mucormycosis has steadily increased in the last two decades (Prista Leão et al., 2020; Garre, 2022), and was exacerbated by the emergence of COVID-19-associated mucormycosis during the SARS-CoV-2 pandemic (Arjmand et al., 2022), particularly in countries such as India, Pakistan, Iran, France, the USA and Brazil (Muthu et al., 2021; Singh et al., 2021; Hoenigl et al., 2022). Therapeutic options for mucormycosis are limited, posing significant challenges for disease management. Antifungals considered effective against mucormycetes include the polyene amphotericin B (AMB), the midlength-tailed azole isavuconazole (IVZ), and the long-tailed azoles posaconazole (PCZ) and itraconazole (ITZ) (Cornely et al., 2019; Vitale et al., 2012). Current guidelines recommend liposomal AMB as a first-line treatment, while PCZ or IVZ are recommended for salvage therapy or for patients with comorbidities such as renal impairment (Cornely et al., 2019; Tissot et al., 2017).

Azole antifungals target sterol 14α-demethylase (Cyp51), a member of the P450 superfamily first purified in 1986 from *Saccharomyces cerevisiae* (Kalb et al., 1986). Cyp51 plays an essential role is the demethylation of lanosterol (Yoshida and Aoyama, 1984), a process requiring three oxidation steps to produce 4,4-dimethylcholesta-8,14,24-trien-3β-ol. This intermediate is subsequently processed into ergosterol, a critical component of fungal cell membranes (Lepesheva et al., 2008; Nes, 2011). Cyp51 enzymes exhibit varying substrate specificities, including lanosterol, eburicol, and obtusifoliol. Azoles act as competitive inhibitors by binding tightly to the heme iron in the active center of Cyp51, thereby reducing ergosterol synthesis. This inhibition not only depletes ergosterol for membrane biosynthesis, but leads to the accumulation of fungistatic C14-methylated intermediates and toxic diols (e.g., 14-methylergosta-8,24(28)-dien-3,6-diol, 14-methylergost-8-en-3,6-diol). These disruptions compromise membrane fluidity and integrity, activate cellular stress responses, and inhibit fungal growth (Kelly et al., 1995; Kelly et al., 1997; Vanden Bossche, 1991; Pérez-Cantero et al., 2020).

Mucorales are intrinsically resistant to short-tailed azoles such as fluconazole (FCZ) and voriconazole (VCZ) but remain susceptible to the long-tailed azole PCZ (Sagatova et al., 2016; Caramalho et al., 2017). *In silico* analyses have suggested that the evolutionarily conserved Y129F and V291A amino acid substitutions in the substrate binding pocket of Cyp51-F5 isoform of mucormycetes confer resistance to FCZ and VCZ, without significantly affecting susceptibility to PCZ (Caramalho et al., 2017).

Acquired mutations in the Cyp51 B-C loop of other human pathogenic fungi that are structurally aligned with MluCyp51-F5 Y129F have been associated with resistance to FCZ and VCZ but not PCZ. Examples include *Candida albicans*, *Candida parapsilosis*, and *Candidozyma auris* Erg11 Y132F (Rosam et al., 2020). Crystal structures of *S. cerevisiae* Erg11 Y140F/H mutants reveal that resistance to short-tailed azoles likely arises from the loss of a water-mediated hydrogen bond network between Y140 and the tertiary alcohol group in FCZ or VCZ, a feature absent in interactions with long-tailed azoles like ITZ and PCZ (Hargrove et al., 2017). In addition, the V291A substitution in helix I is positioned within the substrate-binding pocket near the 2,4-difluorophenyl ring of FCZ and VCZ, as well as the hydrophobic tails of natural substrates such as lanosterol (Caramalho et al., 2017; Rosam et al., 2020; Castanheira et al., 2020). These structural changes collectively explain the selective resistance to short-tailed azoles while maintaining susceptibility to long-tailed azoles.

This study is the first to investigate the individual *CYP51* genes of *M. lusitanicus* expressed in a heterologous model. This approach allows for the focused examination of target gene products and their amino acid substitutions without interference from other cellular processes such as drug efflux pumps that are present in a homologous model. Furthermore, this study experimentally tests the *in silico* hypothesis that the isoforms MluCyp51-F1 Y130 and V294, as well as MluCyp51-F5 F129 and A293, determine differential affinity of these antifungal targets for short-tailed azoles. Notably, the primary sequence features of the B-C loop and helix I regions in mucormycete Cyp51-F5 appear more closely related to the intrinsically azole-resistant Cyp51s found in plant species than to the sequences observed in MluCyp51-F1.

Our *S. cerevisiae* host, engineered to minimize drug efflux, provides a hypersensitive model for studying the function of individual MluCyp51 isoforms and the effects of amino acid substitutions in the substrate-binding pocket (Ruma et al., 2022; Sagatova et al., 2015). The host strain, AD∆∆, is deleted of seven ABC transporter genes, the *PDR3* transcriptional regulator gene, and the *URA3* and *HIS1* genes. Additionally, the *pdr1-3* gain of function mutation enables constitutive overexpression of the target gene via the *PDR5* promoter. This model has been used to investigate the role of other fungal *ERG11*/*CYP51* genes for both molds and yeasts, especially basidiomycetes and ascomycetes (Ruma et al., 2022; Keniya et al., 2018; Keniya et al., 2013; Toepfer et al., 2023; Suh et al., 2006; Spatafora et al., 2016).

The *GAL1* promoter has been used as a backup strategy to address the significant phylogenetic distance between Mucorales and Saccharomycetales. Native Erg11 expression, which encodes the essential endogenous Cyp51, can be activated or deactivated by using galactose or glucose as the carbon source (Ruma et al., 2022).

NADPH-cytochrome P450 reductase (CPR) facilitates electron transfer from NADPH to the heme domain of Cyp51 via its flavin adenine dinucleotide (FAD) and flavin mononucleotide (FMN) binding domains (Hannemann et al., 2007; Murataliev et al., 2004). Given the significant evolutionary distance between mucormycetes and *S. cerevisiae*, incompatibility might arise between the endogenous yeast CPR and heterologously expressed mucormycete Cyp51s. Cognate CPRs have been shown to more effectively support Cyp51 activity (Hosseini et al., 2024; Keniya et al., 2018; Venkateswarlu et al., 1998).

Our hypothesis has been validated using *S. cerevisiae* strains expressing recombinant MluCyp51-F1, MluCyp51-F5 and MluCyp51-F5 variants (MluCyp51-F5 F129Y, MluCyp51-F5 A293V, and MluCyp51-F5 F129Y A293V) from the *PDR5* locus and the cognate recombinant MluCPR co-expressed from the *PDR15* locus under the control of the PDR5 promoter. The constructs were verified through growth on selective media, colony PCR, Sanger DNA sequencing, SDS-PAGE, Western blotting, and nano LC-MS/MS analysis of tryptic fragments. Strain fitness was assessed using growth kinetics in glucose media, while respiratory capacity was evaluated in glycerol-based media. Drug susceptibilities were determined using broth microdilution antifungal susceptibility assays in accordance with EUCAST guidelines (EUCAST, 2023), with media adapted to support yeast growth. The effects of azole exposure on ergosterol biosynthesis were analyzed by GC-MS. In addition, three-dimensional *in silico* modelling of both MluCyp51 isoforms was performed. Comparison of X-ray crystallography structures of Cyp51s bound to lanosterol, a short-tailed azole (FCZ) and a long-tailed azole (PCZ) provided insight into the biochemical organization of the substrate binding pocket.

## Materials and methods

### Selection of open reading frames

In order to identify putative MluCyp51-F1, MluCyp51-F5 and MluCPR open reading frames (ORF), *in silico* analysis was performed using the full genome sequence translation database for *M. circinelloides* f. *lusitanicus* MU402 strain derived from CBS277.49 (Sagatova et al., 2018), using the corresponding protein sequence from *R. arrhizus* (*R. delemar* RA 99–880) as described in previous studies (Sagatova et al., 2016; Caramalho et al., 2017; Venkateswarlu et al., 1998; Keniya et al., 2014; Warrilow et al., 2019; Lah et al., 2008; Zulak et al., 2018; Macedo et al., 2018; Riley et al., 2015; Novak et al., 2015) as a reference. ORFs with best positive alignment scores were selected for back-translation (amino acid to nucleic acid) with codon optimization and modifications needed for the expression in the heterologous *S. cerevisiae*.

### Selection of MluCyp51-F1

*Rhizopus delemar* RA 99–880 lanosterol 14α-demethylase protein (Sequence ID: XP_067522475.1) was used as a reference. Based on the positive alignment score (NCBI BLAST) cytochrome P450 CYP51 (*M. circinelloides* REF sequence ID: EPB89264.1) was the closest match: (81% identities, 90% positives). This MluCyp51-F1 primary sequence was used to obtain the codon optimized ORF.

### Selection of MluCyp51-F5

*R. delemar* RA 99–880 protein RO3G_16595 (Sequence ID: EIE91884.1) was used as a reference. Based on the positive alignment score (NCBI BLAST) cytochrome P450 CYP51 (*M. lusitanicus* CBS 277.49 sequence ID: OAD07570.1) was the closest match: (76% identities, 84% positives). This MluCyp51-F5 sequence was used initially to obtain a codon optimized ORF synthesized by ATUM (Newark, CA, United States). Subsequent analysis of related sequences (e.g., EPB84539.1) led to the conclusion that sequence ID: OAD07570.1 artificially truncates the *N*-terminal part of this protein. The DNA sequence upstream of the published start codon contained an open reading frame extending a further 21 codons including a start ATG. The translated polypeptide closely matched *R. ahrrizus* Cyp51-F5 and was therefore codon optimised and used to extend the existing coding sequence. The resultant score for full length alignment these ORFs was 75% identities and 84% positives.

### Selection of MluCPR

Selection of the *CPR1* gene required more in-depth analysis. The well characterized genome of *S. cerevisiae* contains a single gene encoding NADP-cytochrome P450 reductase (NP_011908.1, 691 AA), but there are two putative CPR ORFs in *R. ahrrizus*: A (EIE89541.1, 713 AA) and B (EIE77771.1, 708 AA), and three in *M. lusitanicus* CBS 277.49: A (OAD00099.1, 648 AA), B (OAC97849.1, 646 AA) and C (OAD05985.1, 617 AA). Both CPRs from *R. ahrrizus* are closely related (88% positives) and have modest similarity scores against *S. cerevisiae* Cpr1 (56 and 57% of positive protein alignment respectively). The longer RaCPR isoform A was chosen as a reference.

The relative similarity of three ORFs from the *M. lusitanicus* CBS 277.49 strain was assessed using the NCBI protein blastp alignment algorithm with conditional compositional matrix adjustment. The analysis indicates that isoforms A and B are closely related (80% identities), and that isoform C is a truncated version (62 and 60% identity to isoforms A and B, respectively). We noted that all three published ORFs from *M. lusitanicus* CBS 277.49 were *N*-terminally truncated compared with the CPRs from related organisms such as *M. circinelloides* f. *circinelloides* 1006PhL EPB87224.1713 AA. Analysis of the raw published gene sequence upstream of the proposed starting codon located an in frame stretch of 195 nucleotides that encoded an additional 55 amino acid sequence. This extended the A isoform to a full length of 713 amino acids, comparable to *R. arrhizus isoform* A. The resultant MluCPR isoform A ORF was the closest match to the reference RaCPR isoform A (80% identity, 90% positives) and was chosen for codon optimization and expression.

### Codon optimization and modification of selected genes

The selected protein sequences of MluCyp51-F1, MluCyp51-F5 and MluCPR were back-translated to nucleotide sequences and codon optimized for *S. cerevisiae* using ATUM’s (Newark, CA, United States) patented in-house algorithms designed to harmonize codon frequencies, avoid unwanted restriction sites and secondary structures. Sequences suggested by ATUM were also audited for rare (<9%) codons/bottlenecks by applying the Graphical Codon Usage Analyzer^1^ tool (Graphical Codon Usage Analyser, 2019) to plot sequential relative efficiency for each codon using the codon usage table for *S. cerevisiae* (Codon Usage Database, 2019). Similar plots for the corresponding native genes of yeast (Sc*CPR*, Sc*ERG11*) were used as a reference. Instances where the same nucleotide was repeated at least seven times were replaced with appropriate synonymous codons to prevent unwanted secondary structures and sequencing difficulties. The upstream sequence CTCGTTCGAAAGACTTAATTAAAAA (*PDR5* promoter) and downstream sequence GGCGGCCGCCATCATCACCATCATCATTAA (GGR flexible linker, His-Tag and STOP-codon) were added for universal priming convenience, recombinant protein detection and purification. The recombinant sequences synthesized by ATUM were supplied as inserts in the high-copy plasmids, with a kanamycin resistance marker suitable for expression in *Escherichia coli* (Supplementary Figure S1). An exception was the 21-codon extension of MluCyp51-F5. This was codon harmonized with the host strain by means of codon usage tables for *M. circinelloides* f. *lusitanicus* and *S. cerevisiae* obtained from Codon Usage Database.^2^ For each degenerate codon, relative codon abundance for *Mucor* was closely matched with that of *S. cerevisiae*. The resultant sequence was synthesized as a 110-mer primer (metabion international AG, Planegg, Germany) and attached to the original ORF by PCR.

### Strains and growth media

The parental and mutant strains used in this study are shown in Supplementary Table S1. Media were prepared and cells cultured according to Keniya et al. (2018). YPD media consisted of 1% yeast extract, 2% peptone (Formedium, Norfolk, United Kingdom), 2% glucose (Carl Roth GmbH + Co. KG, Karlsruhe, GER) and 1.8% agar (VWR International, Radnor, PA, United States). For conditional expression of the native Sc*ERG11* controlled by the *Gal1* promoter in the AD∆∆Gal host and its derivatives, the glucose was replaced with 2% galactose (Formedium, Norfolk, United Kingdom). Transformants were selected on agar plates containing synthetic media (SD): 0.67% (w/v) yeast nitrogen base without amino acids (Formedium), 10 mM MES and 20 mM HEPES buffered to pH 6.8 with 1 M TRIS (Carl Roth GmbH + Co. KG, Karlsruhe, GER), together with either uracil or histidine dropout supplement (Formedium, Norfolk, UK). For the *S. cerevisiae* constructs (Keniya et al., 2018), the RPMI media suggested by EUCAST guidelines for susceptibility testing was replaced with liquid SD media buffered to pH 6.8. For sterol analysis, liquid Sabouraud media containing 1% peptone prepared from casein (Sigma-Aldrich, Darmstadt, GER) and 4% glucose (Carl Roth GmbH + Co. KG, Karlsruhe, GER) was used. To exclude petite mutants, positive transformants was checked for their ability to growth on YP glycerol plates (1% yeast extract, 2% peptone, 3% glycerol, 1.8% agar) and to reduce triphenyltetrazolium chloride (TTC) (Goldring et al., 1970; Ogur et al., 1957).

### Chemicals and kits

Desalted oligonucleotides (Supplementary Table S2, metabion international AG, Planegg, GER) were used at a final concentration of 0.5 μM in a 50 μL reaction. Transformation cassettes were amplified according to manufacturer’s instructions using Phusion^®^ High-Fidelity PCR Master Mix with HF Buffer (New England BioLabs (NEB), Ipswich, MA, United States) using a Peqlab peqSTAR 2X thermocycler (Peqlab, Erlangen, GER). Transformation cassettes were gel purified using NucleoSpin Gel and PCR Clean-up kits (Macherey-Nagel, Düren, GER). Transformation was carried out using the Alkali-Cation^™^ Yeast Transformation Kit (MP Biochemicals, Irvine, CA, United States) according to the manufacturers protocol, with the incubation in lithium acetate reduced to 20 min. Colony PCR used Q5^®^ High-Fidelity 2X Master Mix (NEB, Ipswich, MA, United States) to detect correct integration into the *S. cerevisiae* genome. Genomic DNA was extracted using the Yeast DNA Extraction Kit (Thermo Fisher Scientific^™^, Waltham, MA, United States) and DNA sequences of full length MluCyp51 isoforms and MluCPR were obtained using the Mix2Seq kit (Eurofins, Luxemburg, LUX) and sequenced by Eurofins. For protein content determination, the DC^™^ Protein Assay Kit II (BioRad, Feldkirchen, GER), was used.

### Antifungals

The antifungals used for susceptibility testing, growth kinetic studies and sterol analysis, VCZ (PZ0005), FCZ (F8929), PCZ (SML2287), ITZ (PHR1834) and AMB (A2411), were purchased from Merck (Darmstadt, GER). IVZ was obtained from Pfizer (Berlin, GER).

### Construction of recombinant strains

Use of the AD∆∆ host strain has been reported in several publications (Sagatova et al., 2016; Sagatova et al., 2015; Keniya et al., 2018; Lamping et al., 2007). The construction of heterologous strains was carried out according to the transformation strategy described by Keniya et al. (2018). Codon-optimized ORFs were amplified from plasmids (ATUM, Newark, CA, United States), using primers pABC3-PacI-F and Not1-6x His-R (see Supplementary Table S2). MluCyp51-F1 and MluCyp51-F5 ORFs were fused with suitable flanking polynucleotides of the *PDR5* locus (upstream region amplified with primers PDR5F/pABC3-PacI-R, downstream region with Not1 6x His-F /PDR5 288DS-R). MluCPR was fused with flanking regions of PDR15 locus (upstream amplified by MRP20-697ORF-F/pABC3-PacI-R and downstream with Not1 6x His-F, PDR15DS-R). The three components of transformation cassettes were fused by PCR on a 1:1:1 molar ratio using primers PDR5F and PDR5 288DS-R for Cyp51 isoforms and PDR15US-F & PDR15DS-R specific for the *PDR15* locus (see Supplementary Table S2). One microgram of the transformation cassette was transformed into the host strains and integrated in the specific locus by homologous recombination. The full-length MluCyp51 isoform cassettes targeted to the *PDR5* locus carried a linker sequence (GGR), a *C*-terminal hexa-histidine (His_6_), a *PGK1* transcription terminator and *HIS1* selection marker (see Supplementary Figures S1, S2). For several strains, the His_6_-tagged cognate MluCPR was incorporated into the *PDR15* locus together with the *PDR5* promotor. For transformation into the *PDR15* locus, the *URA3* gene incorporated in the transformation cassette downstream of the *PGK1* transcription terminator was used as selection marker instead of *HIS1* (see Supplementary Figure S2).

### Colony PCR

The DNA of selected transformants was amplified by colony PCR in a total reaction volume of 15 μL using PDR5F/PDR5 288DS-R, PDR15US-F/ PDR15DS-R as primer pairs for *PDR5* and *PDR15* loci, respectively, (see Supplementary Figure S3). PCR conditions were 98 °C for 20 s initially, followed by 35 cycles (95 °C, 20 s, 55 °C 20 s, 68 °C 1 min/kb) and an additional elongation step at 68 °C for 10 min. The reaction products were visualized after electrophoresis in 0.6% agarose gels (LE agarose, Biozym, Hessisch Oldendorf, GER) using a GelDoc (BioRad, Feldkirchen, GER) and Midori Xtra (NipponGenetics, Düren, GER) as dye.

### Sequence verification

Heterologous genes and their sites of integration were verified by sequencing from 758 nucleotides upstream and 41 nucleotides downstream of the MluCyp51-F1 and MluCyp51-F5 transformation cassette integration sites in the *PDR5* locus, using the primers PDR5-F-v2, PDR5 US 126-F, PGK1-R, ScHIS1-ORF27-F, MluF5 637-F, pABC3-PacI-F primers for their *PDR5* integration locus. The MluCPR sequence confirmation covered 1,033 nucleotides upstream and 41 nucleotides downstream of the ORF, using PDR5 US 126-F, MluCPR ORF 701-F, PGK1-R, ScUra ORF322-R, pABC3-PacI-F, primers for its *PDR15* integration locus (see Supplementary Figure S2).

### Preparation of crude membranes expressing His-tagged proteins and Western blot analysis

Small- and large-scale crude membrane preparation were obtained as previously described by Madani et al. (2021) and Monk et al. (2014), respectively. The protein content of the crude membrane fraction was determined with Bovine serum albumin (Thermo Fisher Scientific^™^, Waltham, MA, United States) as standard. Samples of 20 μg of protein were separated by SDS-PAGE on 8% acrylamide gels according the method of Laemmli (1970) and either stained with Coomassie blue R250 or electro-transferred onto nitrocellulose membranes (BioRad, Feldkirchen, GER) using the Trans-Blot Turbo Transfer System (BioRad, Feldkirchen, GER) according to a standard protocol at 25 V for 30 min. Color Prestained Protein Standard, Broad Range (10–250 kDa, BioRad, Feldkirchen, GER) served as a size standard for SDS PAGE and Western blots. His_6_-tagged proteins were decorated with a conjugated antibody (Anti-His_6_-peroxidase from mouse, Merck, Vienna, AUT) and antigen–antibody complexes detected by ECL using the ImageQuant^™^ LAS 4000 Imaging System (GE Healthcare, Tiefenbach, AUT) (Keniya et al., 2018). For evaluation of relative expression, crude membranes from the host strain AD∆∆ provided the negative control and crude membranes from strains strain Y941 and Y2300 constitutively expressing *S. cerevisiae* Erg11 from the *PDR5* locus served as positive controls. Strain Y941 retains the native Sc*ERG11* while it is deleted in Y2300 (Supplementary Table S1). The native Erg11 in AD∆∆ lacks a His_6_ tag and is not detected in the Western blot. Native Erg11 had a calculated size of 60.7 kDa based on its amino acid sequence. A constitutively expressed Coomassie R250-stained tubulin-like protein band (49 kDa on SDS-PAGE) was used to ensure comparable loading of crude membrane protein.

### Spot susceptibility assays

Strains were grown in liquid culture (YPD, YPDgal for MluCPR) until they reached exponential growth (OD_600_ = 0.6). From the original cultures, a serial dilution was prepared until 10^−3^. Five μL of each dilution step was then spotted onto pre-warmed agar (YPD, YPDgal) containing either no drug, 1 μM VCZ or 1 μM PCZ. Plates were then incubated for 48 h at 30 °C and images acquired using the Fusion absolute (Vilber, Eberhardzell, Germany).

### Identification of recombinant proteins by mass spectrometry of trypsin digested fragments

Coomassie-stained bands were excised from the SDS-PAGE gel, reduced with dithiothreitol, alkylated with iodoacetamide and digested with trypsin as previously described (Faserl et al., 2019). Tryptic digests were analyzed using an UltiMate 3000 RSCL nano-HPLC system (Thermo Fisher Scientific^™^, Waltham, MA, United States) coupled to an Orbitrap Eclipse mass spectrometer (Thermo Fisher Scientific^™^) via a Nanospray Flex ionization source (Thermo Fisher Scientific^™^). The mass spectrometer was equipped with a high-field asymmetric ion mobility spectrometer interface and operated in the data dependent mode with compensation voltages of −45 V and −65 V and a one second cycle time. Survey full scan MS spectra were acquired from 375 to 1,500 *m*/*z* at a resolution of 240,000, with an isolation window of 1.2 mass-to-charge ratio (*m*/*z*), a maximum injection time (IT) of 50 ms, and an AGC target of 400,000. The MS2 spectra were measured in the Orbitrap analyzer at a resolution of 15,000 with a maximum IT of 22 ms and an AGC target of 50,000. Selected fragment patterns were refragmented by higher-energy collisional dissociation with normalized collision energy of 28%.

Data analysis was performed using Proteome Discoverer 2.5 (Thermo Fisher Scientific^™^) with the Sequest search engine. The raw files were searched against the yeast database (orf_trans_all) with the amino acid sequences of MluCyp51-F1, MluCyp51-F5 and MluCPR added to the FASTA file. Precursor and fragment mass tolerance was set to 10 ppm and 0.02 Da, respectively, and up to two missed cleavages were allowed. Carbamidomethylation of cysteine was set as static modification and oxidation of methionine was set as variable modification. Acetylation, methionine-loss, and methionine-loss plus acetylation were set as *N*-terminal dynamic modification of proteins. Peptide identifications were filtered at a 1% false discovery rate.

### Purification of recombinant protein and drug binding assay

His_6_-tagged recombinant proteins were purified by extracting crude membranes using *n*-decyl-*β*-D-maltoside as detergent, Ni-NTA-agarose affinity chromatography (Qiagen, Venlo, NED) and size-exclusion centrifugation using Amicon Ultra-4 membrane filters, (Cutoff 50 kDa MVCO, Merck Millipore, Darmstadt, GER) as described by Sagatova et al. (2015). To evaluate Type II binding, the affinity-purified protein (final concentration 5.4 μM) was measured with either VCZ or PCZ (final concentration of 40 μM) from 250 to 600 nm at 1 nm intervals (Ruma et al., 2022) using Varioskan Lux microplate reader (Thermo Fisher Scientific^™^, MA, United States).

### Growth rates in liquid culture

A single colony was cultured in 3 mL YPD media at 30 C and 200 rpm in a horizontal shaker (InFors AG, Bottmingen, CH), the OD_600nm_ measured after 16 h (NP80, Implen GmBH, Munich, GER) and 200 μL cultures inoculated at OD_600nm_ = 0.1 in a 96-well plate (Greiner Bio-One, St. Gallen, CH). The plate was incubated at 30 °C and 240 rpm and the OD_600nm_ with media blank substracted measured every 30 min for 14 h using a Varioskan Lux microplate reader (Thermo Fisher Scientific^™^, MA, United States). To measure the effect of antifungals, the media was supplemented with PCZ (0.025 μM and 0.1 μM) or VCZ (0.1 μM and 1.0 μM). All strains were tested in three biological and two technical replicates, respectively. Specific growth rates (*μ*) and doubling times (*tD*) were calculated during exponential growth using the formulas $\mu =\frac{\ln(X(t):X(0))}{\left(t-t0\right)}$ and $\mathrm{tD}=\frac{\ln(2)}{\mu }$ , where *t* is the time point and *X* the optical density.

### Drug susceptibility phenotypes

Susceptibility testing was performed according to the EUCAST guidelines (v 7.3.2, 22 April, 2020) EUCAST (EUCAST, 2023) in SD complete media using commercially available azole drugs and AMB. Growth was determined by measuring OD_450nm_ using a Tecan Sunrise spectrophotometer (Tecan Trading AG, Männedorf, CH) after incubation at 30 °C for 48 h. Minimal inhibitory concentration (MIC) was defined at 50% inhibition for azoles and 90% for AMB compared to the no drug growth control. Each antifungal construct was tested in three biological replicates. For fold change calculations, the mean MIC of individual strains were divided by the mean MIC of the AD∆∆ strain.

### Sterol content of *Saccharomyces cerevisiae* strains

Overnight cultures were shaken at 240 rpm for 16 h in 20 mL Sabouraud media at 30 °C using a horizontal shaker (Infors AG, Bottmingen, CH). To ensure cells were in log phase on exposure to antifungal treatment, cell loading densities were adjusted to reach OD_600nm_ = 2 after a further 12 h. Cells harvested by centrifugation were incubated in 50 mL Sabouraud media with either 1.0 μM and 0.1 μM VCZ or 0.1 μM and 0.025 μM PCZ, or no antifungal. After 6 h at 30 °C and 200 rpm, the cells were recovered by centrifugation and shock frozen in liquid nitrogen. The pellet was lyophilized overnight (Goldbrunn^™^ 450, Berlin, GER) and the sterol extraction and gas chromatography–mass spectrometry (GC–MS) analysis carried out according to the method described by Müller et al. (2017).

### Software

Statistical analysis and graph construction used Graphpad Prism 8 (version 8.0.1 for Windows, GraphPad Software, San Diego, CA, United States, www.graphpad.com). ImageJ (version 1.53k, Wayne Rasband and contributors, National Institutes of Health, United States, http://imagej.nih.gov/ij) was used to analyze Western blots. Plasmid Maps were constructed using SnapGene software (Version 5.3.1 Boston, MA, United States, www.snapgene.com). *In silico* three dimensional visualizations were performed using PyMOL^™^ (PyMOL Molecular Graphics System, Version 3.0 Schrödinger, LLC, www.pymol.org) with incorporated substrates (LAN: *6UEZ*, FCZ: *4WMZ*, *PCZ*: *6E8Q*) from Protein Data Bank (PDB, www.wwpdb.org). Supplementary Figures S2, S4 were created using biorender.com.

## Results

### Successful expression of MluCyp51 isoforms and cognate MluCPR confirms functional proteins and verifies MluCyp51-F5 F129-mediated resistance to short-tailed azoles

**Figure 1:**
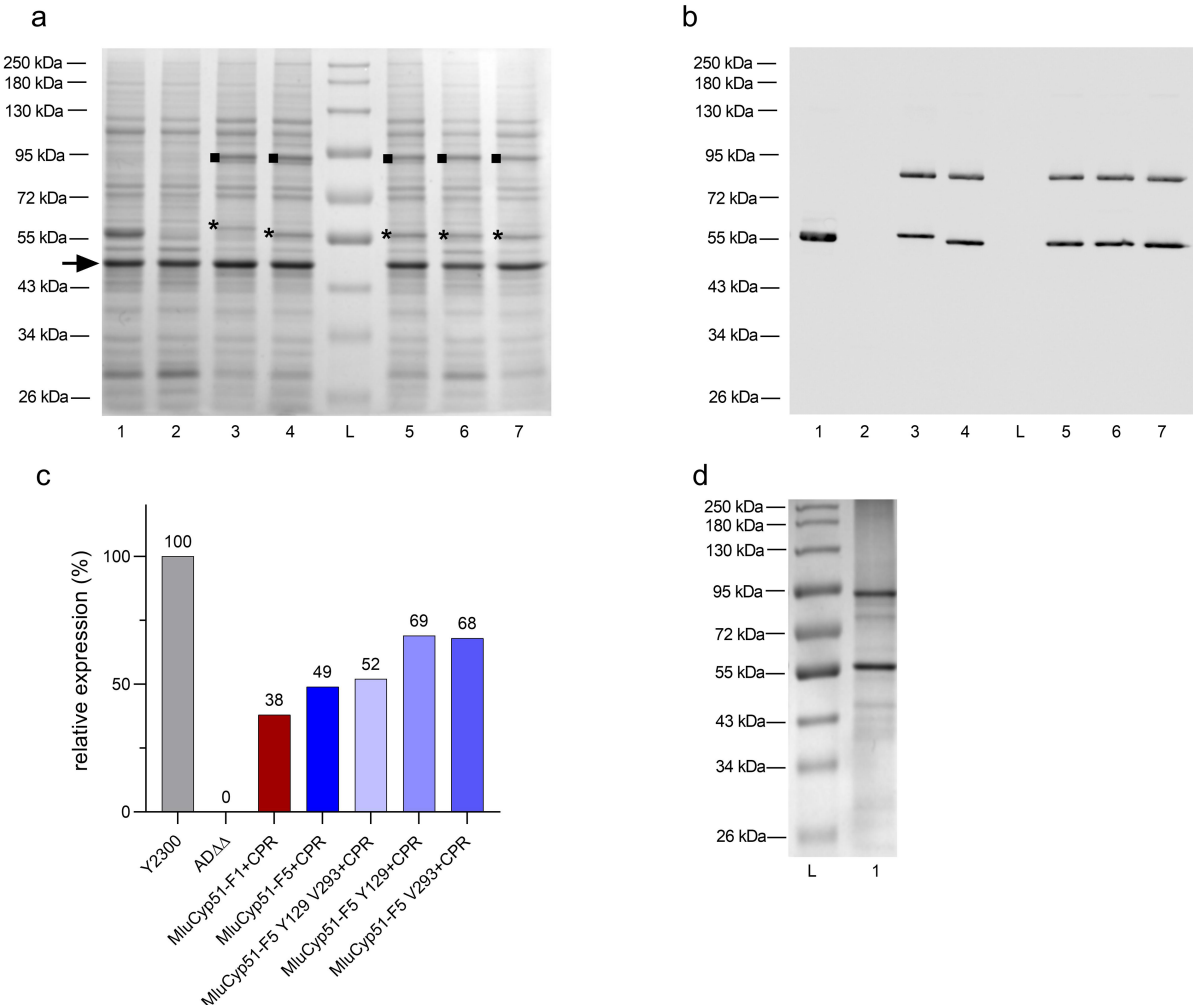
Verification of heterologous protein expression by SDS-PAGE and Western blot. **(a)** SDS-PAGE analysis of the host AD∆∆ strain, positive control, and recombinant strains expressing MluCyp51 isoforms, MluCPR, and MluCyp51-F5 variants co-expressed with CPR. Crude membranes were extracted from glucose-grown strains, and protein samples (10 μg) were separated on 8% acrylamide gels and stained with Coomassie Brilliant Blue R-250. Lane L: Protein Broad Range Standard, Lane 1: Y2300, Lane 2: AD∆∆, Lane 3: MluCyp51-F1 + CPR, Lane 4: MluCyp51-F5 + CPR, Lane 5: MluCyp51-F5 F129Y A293V + CPR, Lane 6: MluCyp51-F5 F129Y + CPR, Lane 7: MluCyp51-F5 A293V + CPR. The highly expressed tubulin-like protein (MW = 49 kDa, marked with an arrow) was used for normalization. The asterisk (*) indicates the recombinant Cyp51 isoform, and the square (■) marks the CPR. **(b)** Western blot analysis of control and recombinant strains. Bands correspond to MluCyp51 variants and CPR. **(c)** Relative protein expression levels of recombinant strain Cyp51 normalized to the control strain Y2300 and the tubulin-like protein. **(d)** SDS-PAGE after Ni-NTA affinity purification of MluCyp51-F5 + CPR. Recombinant protein bands expressed from the *PDR5* and *PDR15* loci were excised, and their identities were confirmed by nano LC-MS/MS analysis of trypsin-digested peptide fragments, achieving primary sequence coverages of 61–69% (Supplementary Table S4). The molecular weights of MluCyp51-F1 and MluCyp51-F5, based on their amino acid sequences (Supplementary Table S4), are 59.0 kDa, with MluCyp51-F1 migrating at ~60 kDa and MluCyp51-F5 at ~57 kDa on SDS-PAGE. The recombinant MluCPR exhibited a band with a relative molecular weight of 92 kDa, consistent with its calculated molecular weight of 81.8 kDa.

Crude membranes from glucose-grown strains were analyzed by SDS-PAGE and stained with Coomassie Brilliant Blue R-250 (Figure 1a). MluCyp51-F1 and MluCyp51-F5, with calculated molecular weights of 59.0 kDa, migrated at ~60 kDa and ~57 kDa, respectively. The recombinant MluCPR appeared at ~92 kDa, consistent with its calculated molecular weight of 81.8 kDa. Recombinant protein bands from the *PDR5* and *PDR15* loci were excised and identified by nano LC-MS/MS of trypsin-digested peptides, achieving 61–69% sequence coverage (Supplementary Table S4).

Suppression of *GAL1* promotor-dependent expression of the endogenous ScErg11 was checked using mass spectrometry. Peptides specific to MluCyp51-F1 or MluCyp51-F5, but not ScErg11, were detected, confirming that Sc*ERG11* expression was suppressed by glucose-induced repression of the *GAL1* promoter (Supplementary Table S5) (Ruma et al., 2022). As lanosterol 14α-demethylase (sterol C14-demethylase) is essential in *S. cerevisiae*, complementation of its absence by heterologous expression of either MluCyp51-F1 or MluCyp51-F5 demonstrated that these proteins are functional and the heterologous expression of evolutionarily distant fungal proteins is feasible (see Supplementary Figure S4).

Western blots decorated with an anti-His_6_ antibody were used to assess the relative expression of the MluCyp51 isoforms and MluCPR (Figure 1b). Protein expression was calculated relative to ScErg11 (100%) expressed by strain Y2300 from the *PDR5* locus (Figure 1c). MluCyp51-F1 and MluCyp51-F5 were expressed from the *PDR5* locus at relative expression levels of 48 and 53%, respectively (see Supplementary Figure S5). Co-expression of MluCPR from the *PDR15* locus reduced the relative expression of MluCyp51-F1 to 38% and MluCyp51-F5 to 49%. MluCPR was expressed at levels equivalent to MluCyp51-F1 and at 79% of the level of MluCyp51-F5 (Supplementary Figure S6). For MluCyp51-F5 Y129 V293 + CPR, MluCyp51-F5 Y129 + CPR, MluCyp51-F5 V293 + CPR, MluCPR expression was at 90, 123, and 81%, respectively.

**Figure 2:**
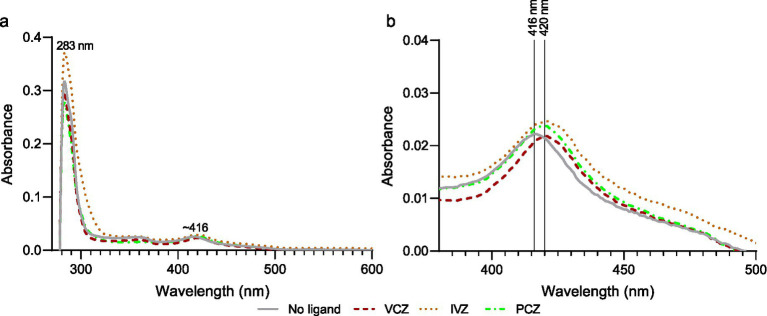
Protein binding analysis of *M. lusitanicus* Cyp51-F5 and NADPH-cytochrome P450 reductase (CPR). **(a)** Absorbance spectrum of 5.4 μM Ni-NTA purified *M. lusitanicus* Cyp51-F5 and CPR. **(b)** Spectral peak shift observed upon incubation with excess (40 μM) voriconazole (red), isavuconazole (orange), or posaconazole (green). Peak maxima are indicated with vertical lines. The annotated peak in **(a)** is at approximately 416 nm.

MluCyp51-F5 and MluCPR from strain SC2347 were partially purified (Figure 1d). Protein folding was evaluated by testing the MluCyp51-F5 in the preparation for Type II binding in the presence of excess VCZ, IVZ, or PCZ according to Ruma et al. (2022). The absolute spectrum of the preparation (Figure 2a) showed peaks at 283 nm (aromatic amino acids) and 416 nm (heme). Incubation of 5.4 μM of the Cyp51 (based on heme content) with 40 μM VCZ, IVZ, or PCZ shifted the 416 nm peak to 420 nm (Figure 2b). This shift, attributed to Type II binding of the triazole group displacing water bound to the heme iron in the substrate-binding pocket, confirmed that the enzyme preparation binds azoles and appears functional, but does not give exact information of the binding affinity or efficacy.

Complementation of the essential endogenous Sc*ERG11* gene, which is supressed in the AD∆∆Gal-based strain during growth on glucose, confirmed proper protein folding, localization and functionality of the recombinant MluCyp51-F1 and MluCyp51-F5 isoforms in the *S. cerevisiae* expression system. Drug susceptibility profiles were assessed for each recombinant strain and appropriate controls for a wide range of azole antifungals currently used systemically, as well as AMB due to its independent mode of action and known cross-resistance. Table 1 summarizes the mean minimal inhibitory concentration (MIC) values in μM obtained for each strain tested (MIC values in mg/L are given in Supplementary Table S6).

**Table 1:** Mean minimal inhibitory concentration (MIC) values (μM) and standard deviation of the heterologous *S. cerevisiae* strains overexpressing the respective MluCyp51 variants.

Strain	AMB	FCZ	VCZ	IVZ	ITZ	PCZ
AD∆∆	1.1 ± 0.0	3.3 ± 0	0.05 ± 0.0	0.04 ± 0.0	0.05 ± 0.0	0.04 ± 0.0
MluCPR	2.2 ± 0.0	2.2 ± 0.9	0.05 ± 0.0	0.02 ± 0.01	0.04 ± 0.02	0.04 ± 0.0
MluCyp51-F1	1.1 ± 0.0	4.4 ± 1.9	0.06 ± 0.02	0.05 ± 0.02	0.01 ± 0.0	0.01 ± 0.0
MluCyp51-F5	0.7 ± 0.3	**139.3 ± 60.3**	**1.43 ± 1.24**	0.16 ± 0.11	0.04 ± 0.02	0.02 ± 0.0
MluCyp51-F1 + CPR	1.8 ± 0.6	34.8 ± 15.1	0.48 ± 0.21	**0.38 ± 0.16**	0.04 ± 0.02	0.02 ± 0.01
MluCyp51-F5 + CPR	2.9 ± 1.2	**835.9 ± 0.0**	**11.45 ± 0.0**	**9.14 ± 0.0**	**0.30 ± 0.1**	0.07 ± 0.02
MluCyp51-F5 F129Y + CPR	**3.2 ± 1.8**	13.1 ± 0.0	0.72 ± 0.0	0.14 ± 0.0	0.06 ± 0.0	0.09 ± 0.0
MluCyp51-F5 A293V + CPR	1.6 ± 0.9	52.2 ± 0.0	**1.43 ± 0.0**	**0.29 ± 0.0**	**0.18 ± 0.0**	**0.12 ± 0.04**
MluCyp51-F5 F129Y A293V + CPR	0.5 ± 0.0	3.3 ± 0.0	0.17 ± 0.0	0.04 ± 0.0	0.03 ± 0.0	0.04 ± 0.0

Statistical analysis was conducted using one-way ANOVA, with significant differences compared to the AD∆∆ strain highlighted in bold. An overview of the strains and their respective inserts is provided in Supplementary Table S1.

**Figure 3:**
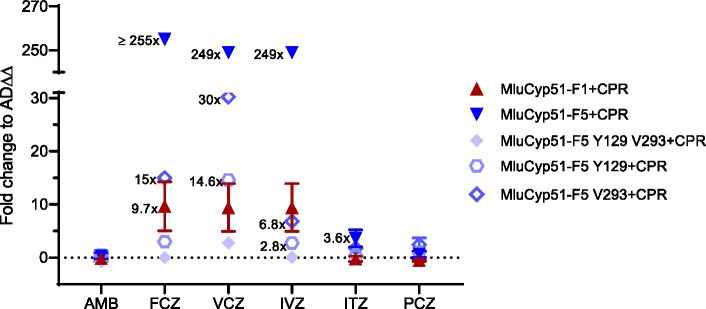
Fold changes in AMB and azole susceptibility. Values of strains expressing recombinant MluCyp51 variants and cognate CPR were compared to the host strain AD*∆∆*. An overview of the strains and inserts is given in Supplementary Table S1.

The strain heterologous expressing MluCyp51-F1 exhibited MIC values comparable to the host strain AD∆∆ for AMB and all azoles tested. In contrast, the strain expressing MluCyp51-F5 showed significantly increased MICs for voriconazole (VCZ, 29-fold) and fluconazole (FCZ, 42-fold), while MIC values for isavuconazole (IVZ), itraconazole (ITZ), posaconazole (PCZ), and AMB remained unaffected compared to AD∆∆ (Table 1 and Figure 3; Supplementary Figure S7). Heterologous expression of MluCPR alone slightly increased MIC values for AMB, but had no effect on azole susceptibility.

Despite lower expression levels of MluCyp51s in strains co-expressing MluCPR, co-expression of MluCPR with MluCyp51-F1 or MluCyp51-F5 further increased MIC values for FCZ, VCZ, and IVZ (~8-fold for MluCyp51-F1, 5- to 50-fold for MluCyp51-F5), and for ITZ (5-fold) in the case of MluCyp51-F5 (Table 1 and Figure 3). Consistent with previous findings (Ruma et al., 2022), these results indicate that the cognate CPR significantly enhances the efficiency of the heterologous expressed Cyp51. Notably, the strain expressing MluCyp51-F5 + CPR exhibited at least 20-fold greater resistance to FCZ, VCZ, and IVZ compared to the strain expressing MluCyp51-F1 + CPR (Supplementary Figures S8, S9). MIC values for ITZ showed a 5-fold increase, while resistance to PCZ was minimal (maximally 5-fold). Amino acid reversions in MluCyp51-F5 + CPR did not significantly alter AMB MIC values for the single substitutions but the double substitution increased susceptibility (F129Y A293V) 6-fold (Table 1 and Figure 3). The strain expressing MluCyp51-F5 F129Y A293V + CPR was fully susceptible to all azole antifungals, including FCZ and VCZ, with MIC values comparable to MluCyp51-F1 + CPR. Single-site reversions (F129Y and A293V) resulted in intermediate increases in susceptibility. For FCZ, VCZ, and IVZ, MIC reductions were at least 9-fold for the A293V substitution and 15-fold for the F129Y substitution. These findings demonstrate that the two amino acid substitutions in MluCyp51-F5 differentially affect the binding of short-tailed azoles, with F129 having a stronger impact on resistance to short- and midlength-tailed azoles. The modest resistance to ITZ was also more affected by the F129Y substitution (3.5-fold) than by the A293V substitution (0.4-fold), while the resistance to PCZ was not significantly modified by either the single or double substitutions.

**Figure 4:**
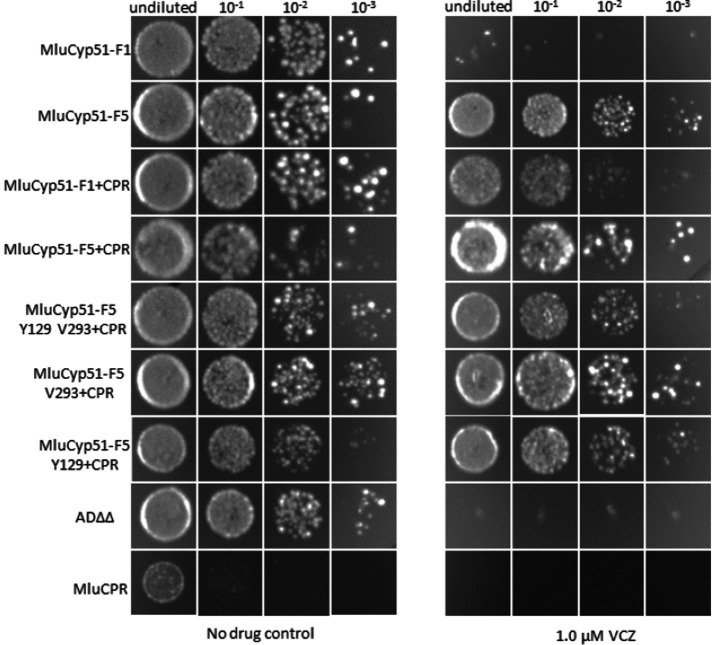
Spot susceptibility assay analysis of strains under no drug conditions and when challenged with 1.0 μM VCZ. For PCZ no growth was detected. See Supplementary Figure S7.

In a complementary experiment (see Figure 4), spot susceptibility assays were performed. Each strain grew in the control medium at all dilution steps, with single colonies observed at higher dilutions. The only exception was MluCPR, which only showed significant growth in the undiluted sample. However, as expected co-expression of MluCPR also enhanced growth for strains expressing MluCYP51-F1 and MluCYP51-F5 under VCZ exposure. When challenged with 1.0 μM VCZ, the MluCyp51-F5 variants grew at all dilution steps, but MluCyp51-F1(+CPR), AD∆∆, and MluCPR did not. Mutation of individual amino acids led to a partial growth in all dilution steps as expected from VCZ MIC values 1.43 μM (MluCyp51-F5 V293 + CPR) and 0.72 μM (MluCyp51-F5 Y129 + CPR), respectively. When both amino acids were modified in MluCyp51-F5 Y129 V293 + CPR, the susceptibility phenotype observed was the same as MluCyp51-F1 + CPR.

### Strain fitness and sterol composition remain stable under heterologous protein expression and VCZ treatment but are affected by PCZ

Strains exhibited good growth, forming crème white, round colonies after 2–3 days (see Figure 4). Growth was comparable to the parental strain on both full media and selective plates when the appropriate selection marker was integrated. As expected for respiration-competent, non-petite cells, all strains tested positive for triphenyltetrazolium chloride (TTC) reduction and growth on glycerol plates was readily seen after 5 days, showing the strains were not petite.

**Figure 5:**
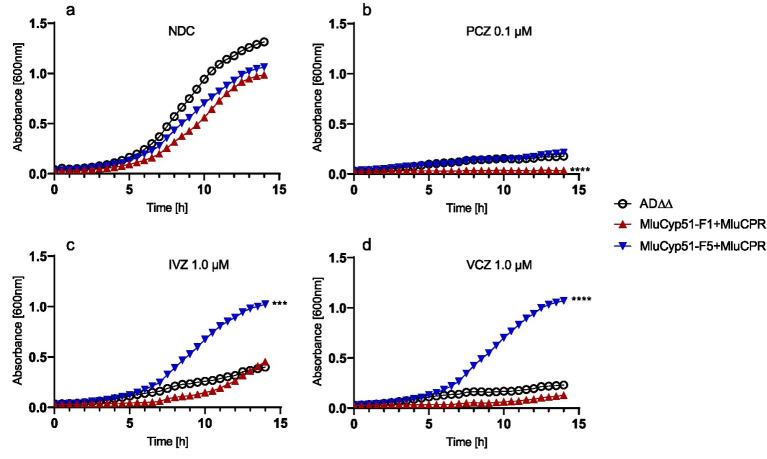
Growth curves of the host strain and strains expressing MluCyp51 isoforms with CPR. Media containing **(a)** no drug—NDC, **(b)** 0.1 μM PCZ, **(c)** 1.0 μM IVZ, and **(d)** 1.0 μM VCZ. Statistical analysis using one-way ANOVA and Dunnett’s *post-hoc* test. Significance levels according to Dunnett’s *post-hoc* test: *p*-value: ^*^p < 0.0332, ^**^p < 0.0021, ^***^p < 0.0002, and ^****^p < 0.0001, *n* = 3. NDC, no drug control. An overview of the strains and inserts is given in Supplementary Table S1.

Growth curves on YPD media (Figure 5a) showed that growth yields and growth rates were not significantly impacted by the heterologous expression of evolutionary distant MluCyp51 and NADPH-cytochrome P450 reductase (MluCPR) genes. Doubling times during exponential growth on glucose (see Supplementary Table S7) ranged from 163 to 173 min for the host strain, MluCyp51-F1 + CPR and MluCyp51-F5 + CPR. These results indicate that constitutive overexpression of MluCyp51-F1, MluCyp51-F5 and MluCPR under control of the *pdr1-3* regulated *PDR5* promoter does not adversely affect growth kinetics.

The fitness of strains expressing MluCyp51 isoforms + MluCPR was challenged based on their azole-susceptibility profiles (Table 1; Supplementary Table S6) by using 0.1 μM (low dose) PCZ, 0.1 μM (low dose) and 1.0 μM (high dose) IVZ, or 0.1 μM (low dose) and 1.0 μM (high dose) VCZ. Under no drug conditions, strains expressing MluCyp51 isoforms with CPR exhibited similar growth rates and yields to the host strain AD∆∆ (see Figure 5a and Supplementary Table S7).

Low-dose PCZ, VCZ and IVZ inhibited the growth of the MluCyp51-F1 + CPR strain. Low-dose PCZ significantly inhibited the growth of MluCyp51-F5 + CPR, while low- and high-dose VCZ and IVZ had no significant effect (Figures 5b–d; Supplementary Figure S10). Under low-dose PCZ treatment (Figure 5b), doubling times increased compared to the no-drug control: 9.5-fold for MluCyp51-F1 + CPR, 2.3-fold for MluCyp51-F5 + CPR, and 2.2-fold for the host strain (Figure 5b and Supplementary Table S7). Growth of the MluCyp51-F1 + CPR expressing strain was completely inhibited by PCZ, and after 14 h, AD∆∆ and MluCyp51-F5 + CPR grew only to OD_600nm_ = 0.26 and 0.36, respectively. Amino acid reversions in MluCyp51-F5 significantly decreased growth yields (Supplementary Figure S10) and further increased doubling times under PCZ treatment (Supplementary Table S7): 11-fold for MluCyp51-F5 F129Y + CPR, 8-fold for MluCyp51-F5 A293V + CPR and 52-fold for MluCyp51-F5 F129Y A293V + CPR.

High dose IVZ inhibited growth of the host strain, while the strain expressing the MluCyp51-F5 isoform was unaffected and the MluCyp51-F1 isoform exhibited an intermediate reduction in growth rate (Supplementary Table S7 and Supplementary Figure S10). After 14 h of exposure, cell densities (OD_600nm_) were 0.39 for the host strain, 0.45 for MluCyp51-F1 + CPR, and 1.0 for MluCyp51-F5 + CPR. Compared to the no-drug control, IVZ treatment increased doubling times by 1.4-fold for the host strain, 1.2-fold for MluCyp51-F1 + CPR, and had no effect on MluCyp51-F5 + CPR. These results align with the MIC of the MluCyp51-F5 + CPR of 9.14 μM for IVZ (Table 1).

The variant strains expressing MluCyp51-F5 F129Y + CPR, MluCyp51-F5 A293V + CPR, and MluCyp51-F5 F129Y A293V + CPR yielded cell densities of 0.79, 0.49, and 0.05, respectively (Supplementary Figure S11) after 14 h exposure to high-dose IVZ. Compared to the no-drug control, doubling times increased by 1.1-fold for MluCyp51-F5 F129Y + CPR, 1.1-fold for MluCyp51-F5 A293V + CPR, and 5.9-fold for MluCyp51-F5 F129Y A293V + CPR (Supplementary Table S7). These results are consistent with the IVZ MIC values of 0.14 μM, 0.29 μM, and 0.04 μM for strains expressing recombinant MluCyp51-F5 F129Y + CPR, MluCyp51-F5 A293V + CPR, and MluCyp51-F5 F129Y A293V + CPR, respectively.

High dose VCZ inhibited the growth of the host strain and MluCyp51-F1 + CPR, but not MluCyp51-F5 + CPR (Figure 5d). After 14 h, final cell densities (OD_600nm_) were 0.30 for the host strain AD∆∆, 0.15 for MluCyp51-F1 + MluCPR, and 1.1 for MluCyp51-F5 + CPR. Corresponding doubling times were 335, 618 and 162 min (Supplementary Table S7). Amino acid reversions in MluCyp51-F5 resulted in varying effects on growth under VCZ treatment (Supplementary Table S7 and Supplementary Figure S11). The strain expressing MluCyp51-F5 A293V + CPR exhibited reduced growth yield (OD_600nm_ = 0.67) and an increased doubling time of 317 min. Similarly, the strain expressing MluCyp51-F5 F129Y + CPR showed a comparable reduction in growth yield (OD_600nm_ = 0.67) but slightly greater susceptibility to VCZ, with a doubling time of 373 min. In contrast, the strain expressing MluCyp51-F5 F129Y A293V + CPR appeared fully susceptible to VCZ, with an OD_600nm_ of 0.05 and a doubling time of 983 min.

These results demonstrate that both Y130 and V294 contribute to the susceptibility of MluCyp51-F1 to VCZ, while F129 and A293 collectively confer VCZ resistance in MluCyp51-F5.

**Figure 6:**
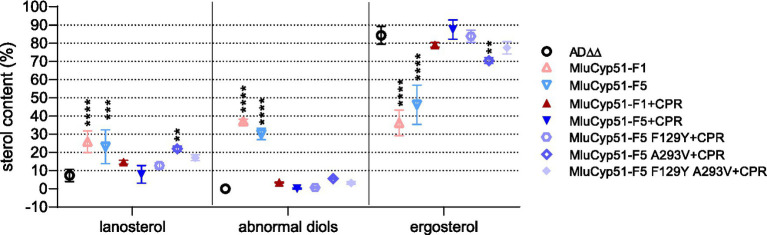
Sterol composition of host and recombinant strains. Sterol composition of host strain AD*∆∆* and strains expressing MluCyp51 variants. Statistical analysis was performed using a two-way ANOVA with Tukey’s *post-hoc* test. Significance levels according to Tukey’s *post-hoc* test: *p*-value: ^*^p < 0.0332, ^**^p < 0.0021, ^***^p < 0.0002, and ^****^p < 0.0001, three biological replicates (*n* = 3). An overview of the strains and inserts is provided in Supplementary Table S1.

The sterol compositions of the parental strain (AD∆∆) and strains expressing either of the two Cyp51 isoforms, with and without the cognate reductase (MluCPR), were analyzed (Figure 6 and Table 2). The detected biosynthesis intermediates were categorized into three groups: lanosterol (precursor and substrate of Cyp51), abnormal diols (14-methylated toxic or fungistatic sterol products due to Cyp51 inhibition: 14-methylergosta-8,24(28)-dienol, 14-methylergost-8-en-3,6-diol), and ergosterol (final product).

**Table 2:** Sterol composition of strains under no drug control (NDC) and azole treatment.

Condition	Sterol	AD∆∆	MluCyp51-F1 + CPR	MluCyp51-F5 + CPR	MluCyp51-F5 F129Y + CPR	MluCyp51-F5 A293V + CPR	MluCyp51-F5 F129Y A293V + CPR
NDC	Lanosterol	7.4 ± 3.4	14.7 ± 1.1	7.9 ± 4.9	12.8 ± 1.5	21.9 ± 1.5	17.1 ± 1.6
	Σ abnormal diols	0.1 ± 0.1	3.5 ± 0.2	0.2 ± 0.3	0.7 ± 0.4	5.5 ± 0.3	3.2 ± 0.8
	Ergosterol	84.3 ± 4.9	79.1 ± 1.3	87.5 ± 5.3	83.9 ± 3.4	70.4 ± 1.6	77.5 ± 3.4
VCZ 0.1	Lanosterol	30.7 ± 6.2	18.8 ± 1.2	8.5 ± 4.3	19.4 ± 1.3	23.6 ± 0.8	25.7 ± 4.5
	Σ abnormal diols	9.0 ± 2.8	5.7 ± 0.4	0.2 ± 0.4	1.5 ± 0.7	6.5 ± 0.2	5.9 ± 2.4
	Ergosterol	59.0 ± 8.6	73.0 ± 0.9	84.2 ± 4.7	77.5 ± 3.4	67.8 ± 1.0	67.1 ± 7.3
VCZ 1.0	Lanosterol	31.0 ± 6.2	25.5 ± 1.1	12.9 ± 6.1	31.5 ± 5.3	26.6 ± 1.3	37.4 ± 6.1
	Σ abnormal diols	9.7 ± 3.1	17.5 ± 1.6	1.2 ± 0.9	5.4 ± 2.6	11.2 ± 0.2	13.3 ± 7.2
	Ergosterol	57.9 ± 9.0	55.1 ± 2.1	82.4 ± 6.2	62.5 ± 8.4	60.8 ± 1.3	48.8 ± 13.3
PCZ 0.1	Lanosterol	31.1 ± 5.8	26.9 ± 1.0	30.7 ± 3.8	39.1 ± 8.8	30.5 ± 0.2	44.2 ± 9.6
	Σ abnormal diols	9.7 ± 3.4	32.9 ± 0.2	29.1 ± 9.5	10.7 ± 9.9	42.6 ± 2.8	22.8 ± 14.0
	Ergosterol	57.9 ± 8.8	38.9 ± 1.2	38.9 ± 12.6	49.7 ± 18.9	26.8 ± 3.0	32.4 ± 23.7

The sterol composition of the host strain (AD∆∆) and recombinant strains (MluCyp51-F1 + CPR and MluCyp51-F5 + CPR) was analyzed under NDC conditions and in media containing VCZ (0.1 μM and 1.0 μM) and PCZ (0.1 μM). The table presents the mean values of lanosterol, abnormal diols, and ergosterol as percentages (% ± SD). Sterols present at levels below 1% were excluded from the table.

In the untreated AD∆∆ host strain, sterol composition as a proportion of total sterols was as follows: 7.4% lanosterol, 0.1% diols, and 84.3% ergosterol. In contrast, strains expressing only MluCyp51-F1 or MluCyp51-F5 (Table 2) showed significant accumulation of lanosterol (25.9, 23.1%, respectively) and of fungistatic/toxic sterols (38.5, 30.4%, respectively) with a corresponding reduction in ergosterol content (36.2% for MluCyp51-F1 and 46.2% for MluCyp51-F5) (Supplementary Table S8).

Co-expression of MluCPR with MluCyp51-F1 or MluCyp51-F5 restored ergosterol production to levels comparable to the AD∆∆ strain (79.1% for MluCyp51-F1 + CPR and 87.5% for MluCyp51-F5 + CPR). Concurrently, the levels of lanosterol and diols were significantly reduced (7.9 and 14.7% lanosterol, and 0.2 and 3.5% diols, respectively). These findings demonstrate the essential role of the cognate reductase in enabling efficient Cyp51 function and restoring normal ergosterol biosynthesis. Based on these results, strains co-expressing MluCyp51 isoforms and MluCPR were used to investigate the effects of azole exposure on sterol biosynthesis.

Table 2 presents the sterol composition of the host strain (AD∆∆), MluCyp51-F1 + CPR, and MluCyp51-F5 + CPR under no drug control (NDC) conditions and when exposed to PCZ (0.1 μM) and VCZ at high (1.0 μM) and low (0.1 μM) doses. These antifungals were selected to represent effective and less effective azole compounds, as determined by MIC values (Table 1) and drug exposure experiments (Figure 5; Supplementary Figure S10). VCZ was chosen as a representative azole for compounds affected by innate resistance, given its similar behaviour to IVZ in these experiments. For the three strains tested (AD∆∆, MluCyp51-F1 + CPR and MluCyp51-F5 + CPR), the MIC values for VCZ were 0.05 μM, 0.48 μM, 11.45 μM, respectively and for PCZ 0.04 μM, 0.02 μM, 0.07 μM, respectively (Table 1). As shown in Table 2, low dose PCZ (0.1 μM) strongly inhibited both MluCyp51 isoforms compared to the NDC. This is evident from the accumulation of lanosterol (14.7% vs. 26.9% for MluCyp51-F1 and 7.9% vs. 30.7% for MluCyp51-F5), abnormal diols (3.5% vs. 32.9% for MluCyp51-F1 and 0.2% vs. 29.1% for MluCyp51-F5) along with a significant reduction in ergosterol content (79.1% vs. 38.9% for MluCyp51-F1 and 87.5% vs. 30.7% for MluCyp51-F5).

Low-dose VCZ (0.1 μM) resulted in a minor increase in lanosterol (4.1%) and toxic sterols (2.2%) for MluCyp51-F1 + CPR strain, while MluCyp51-F5 + CPR remained unaffected (<1%). Ergosterol levels were reduced by 6.1% for MluCyp51-F1 + CPR and 3.3% for MluCyp51-F5 + CPR.

In contrast, high-dose VCZ (1.0 μM) resulted in significant differences between the two strains. In MluCyp51-F1 + CPR, lanosterol increased by 10.8%, diols by 14.0% and ergosterol decreased by 24.0%. For MluCyp51-F5 + CPR, lanosterol increased by 4.4%, diols by 1.0%, and ergosterol decreased by only 5.1%. These results align with MIC tests and drug exposure experiments (Figures 4, 5; Supplementary Figures S9, S10) demonstrating that VCZ exposure disrupts ergosterol metabolism via MluCyp51-F1, but not MluCyp51-F5.

Strains expressing MluCyp51-F5 F129Y + CPR, MluCyp51-F5 A293V + CPR and MluCyp51-F5 F129Y A293V + CPR were challenged with PCZ (0.1 μM) and high dose VCZ (1.0 μM) to assess the impact of individual and combined amino acid substitutions on azole susceptibility (Table 2).

High-dose VCZ (1.0 μM) treatment resulted in concomitant increases in lanosterol and diol content and further reductions in ergosterol production, particularly in the double mutant (MluCyp51-F5 F129Y A293V + CPR) and the F129Y mutant, with a lesser effect observed in the A293V mutant. These changes align well with growth experiments, confirming the impact of these mutations on ergosterol biosynthesis under azole stress.

As expected, PCZ (0.1 μM) treatment significantly increased lanosterol and diol content while reducing ergosterol levels across all mutant strains compared to the NDC. For MluCyp51-F5 F129Y + CPR, lanosterol increased from 12.8 to 39.1%, diols from 0.7 to 10.7%, and ergosterol decreased from 83.9 to 49.7%. For MluCyp51-F5 A293V + CPR, lanosterol increased from 21.9 to 30.5%, diols from 5.5 to 42.6%, and ergosterol decreased from 70.4 to 26.8%. For MluCyp51-F5 F129Y A293V + CPR, lanosterol increased from 17.1 to 44.2%, diols from 3.2 to 22.8%, and ergosterol decreased from 77.5 to 32.4%.

These results confirm that the innate amino acid substitutions in MluCyp51-F5 (F129 and A293) confer resistance to short-tailed azoles. Of these, F129 plays a more significant role than A293 in driving this resistance phenotype.

## Discussion

Heterologous expression of individual proteins of interest in the model *S. cerevisiae* offers a targeted approach that reduces the complexity inherent in homologous systems. This strategy enables focused evaluation of specific metabolic pathways. To explore the innate differential susceptibility of the basal fungus *M. lusitanicus* to azole drugs, selected *M. lusitanicus* genes were integrated into the *PDR5* and *PDR15* loci of the azole-hypersensitive *S. cerevisiae* host strain AD∆∆. This allowed constitutive expression of functional recombinant Cyp51 variants (MluCyp51-F1, MluCyp51-F5) and the cognate NADPH-cytochrome P450 reductase (MluCPR), regulated by the *pdr1-3* gain-of-function transcriptional regulator acting on the *PDR5* promoter (Sagatova et al., 2016). As this was the initial attempt to express MluCyp51 in the phylogenetic distant *S. cerevisiae* host, the *GAL*1 promoter was employed to suppress the expression of the endogenous Sc*ERG11* during growth on glucose. This served as a practical backup strategy designed to ensure successful transformations in case the heterologous *M. lusitanicus* genes failed to function (Ruma et al., 2022). The hypersensitive *S. cerevisiae* model provided a high signal-to-noise environment, facilitating detailed analysis of the phenotypes and biochemistry of individual Cyp51 proteins, including their interactions with key biological and chemical ligands.

The expression of recombinant Cyp51 proteins in the *S. cerevisiae* system has been successfully demonstrated for other closer-related species (Sagatova et al., 2016; Keniya et al., 2013; Toepfer et al., 2023; Lamping et al., 2007; James et al., 2021), and remains a robust method for minimizing background interference from drug efflux pumps. This approach enables structure–function analysis of Cyp51s and their cognate reductases. It has proven effective in addressing fundamental research questions, such as the mechanisms of innate and acquired azole resistance in mucormycetes, and can be extended to less-studied fungal pathogens, including phytopathogens. By elucidating the binding of inhibitory ligands and identifying the importance of amino acid positions F129 and A293 in the MluCyp51-F5 isoform, future research can investigate interactions with novel antifungals and screen for effective drug candidates. The combination of heterologous models with studies in the organism of origin can uncover additional resistance mechanisms, as demonstrated in studies by Navarro-Mendoza et al. (2024) and Nagy et al. (2021). Linking these systems could help identify strategies to improve azole efficacy in medical, agricultural, and veterinary settings.

This study demonstrated that co-expression of the cognate MluCPR with MluCyp51 isoforms significantly enhances Cyp51 function, improving the activity of both MluCyp51-F1 and MluCyp51-F5 isoforms several-fold. MluCPR conferred 10.7- to >255-fold increases in azole resistance and improved ergosterol biosynthesis, as evidenced by reduced lanosterol and toxic diol content and near-normal levels of ergosterol. Furthermore, our data support the idea of an improved substrate turnover in MluCyp51-F5, as this isoform at relatively lower expression levels was able to maintain the highest ergosterol content under no-drug conditions. The recombinant strains created in this study provide opportunities for further *in vitro* analysis of substrate (Type I) and azole inhibitor (Type II) binding efficacy using affinity-purified preparations in carbon monoxide binding assays (Guengerich et al., 2009), kinetic studies with crude membrane preparations, and evaluation of interactions between MluCyp51s and MluCPR. For example, differential fluorescent labeling of MluCPR and MluCyp51s could be used to evaluate the binding preferences of these molecules and interactions with other MluCPR isoforms.

While most fungi, including *R. arrhizus*, possess two CPR isoforms that interact with Cyp51s, exceptions exist, such as *S. cerevisiae* (one CPR) and *Fusarium keratinosum* (four CPR isoforms) (Durairaj et al., 2016). Although the relative benefits of CPR isoforms remain unclear, evidence suggests they may have distinct functional roles, with one isoform contributing to primary and the other to secondary metabolism (Lah et al., 2008; Novak et al., 2015).

This study expands our understanding of the innate resistance of *M. lusitanicus* to short-tailed azoles. Functional expression of recombinant genetically distant MluCyp51 isoforms together with a cognate MluCPR, along with the ability to revert key amino acids in the MluCyp51-F5 isoform, allowed experimental validation of *in silico* predictions originally made for *R. arrhizus* (Caramalho et al., 2017). The resistance of *M. lusitanicus* to short-tailed azoles (e.g., FCZ and VCZ) is primarily attributed to MluCyp51-F5, and its expression conferred at least 26-fold higher resistance than the MluCyp51-F1 isoform, with or without co-expression of MluCPR.

Use of recombinant MluCyp51-F5 variants (MluCyp51-F5 F129Y + CPR, MluCyp51-F5 A293V + CPR and MluCyp51-F5 F129Y A293V + CPR) confirmed that MluCyp51-F5 F129 and A293 are key drivers of resistance to short-tailed azoles. The double mutant conferred susceptibility equivalent to the MluCyp51-F1 + CPR construct while either substitution reduced resistance to FCZ and VCZ by at least 9-fold. The strongest impact on resistance to FCZ and VCZ was due to MluCyp51-F5 Y129, consistent with the role of Y129F substitutions in acquired short-tailed azole resistance in several fungal pathogens (such as *Aspergillus fumigatus*, *Candida* spp., and plant pathogens like *Blumeria graminis* and *Mycosphaerella graminicola*) (Zulak et al., 2018; Mullins et al., 2011; Heick et al., 2017; Pereira et al., 2017; Pandey and Sharma, 2018; Frenkel et al., 2015; Snelders et al., 2015; Wu et al., 2020; Lavergne et al., 2019; Rivero-Menendez et al., 2019; Resendiz-Sharpe et al., 2019).

The importance of MluCyp51-F1 Y130 was expected from *in silico* analysis, as its aromatic hydroxyl forms hydrogen bonds the heme propionate and participates in a water-mediated hydrogen bond network with the tertiary hydroxyl of some azole drugs (Supplementary Figure S11b) (Sagatova et al., 2016; Sagatova et al., 2015). This explains why MluCyp51-F5 confers resistance to fluconazole, voriconazole, and midlength-tailed triazoles like isavuconazole (Supplementary Figure S12b). In contrast, long-tailed triazoles like itraconazole and posaconazole, which lack tertiary hydroxyl groups, do not engage in this hydrogen bond network, and their susceptibility is less affected by the MluCyp51-F5 F129 substitution (Supplementary Figures S11c, S12c).

The slightly weaker contribution of MluCyp51-F5 A293 to resistance may be due to its effect on helix I stiffness and reduced interaction with the phenyl ring of short-tailed azoles. While A293 also reduces binding affinity for long-tailed azoles, this is mitigated by the longer tails of these ligands, which bind along the substrate entry channel. Growth experiments and ergosterol biosynthesis data further support the role of A293 in substrate binding (Supplementary Figures S11b,c, S12b,c) (Hosseini et al., 2024).

Homology modelling suggests that the hydrophobic tail of lanosterol binds parallel to helix I in a crowded region of the active site also bordered by the BC-loop containing Y130 or F129 (Supplementary Figures S11a, S12a). The MluCyp51-F5 V293A substitution may reduce affinity for both azole head groups and natural substrates like lanosterol, a hypothesis that can be tested in Type I binding experiments. In summary, the integration of phylogenetically distant *M. lusitanicus* genes into a *S. cerevisiae* host successfully confirmed that the evolutionary conserved Y129F and V293A substitutions in the MluCyp51-F5 isoform are responsible for intrinsic resistance to short-tailed azoles. This concept has been experimentally validated in *R. arrhizus* and *Rhizopus microsporus* and is likely applicable to other mucormycetes the Cyp51 (Toepfer et al., AAC, under review) of plants and some of their pathogens.

The constructs developed in this study can now be used for biochemical analyses, including kinetic studies, substrate (Type I) and azole (Type II) binding assays, and X-ray crystallography. Structural insights into mucormycete substrate-binding pockets will be invaluable for structure-directed drug discovery. Clinically, the finding of IVZ resistance due to MluCyp51-F5 is concerning, as it may challenge the recent recommendation of isavuconazonium sulfate for treating mucormycosis.

## Data Availability

The sequences have been uploaded to NCBI under the accession number PRJNA3015490.

## Footnotes

^1^ www.GCUA.de

^2^ https://www.kazusa.or.jp/codon/
