# Supplementary material for: Intrinsic resistance to short-tailed azoles in the basal fungus Mucor lusitanicus: functional analysis of Cyp51 isoforms and amino acid substitutions
Source: Front Microbiol. 2025 Nov 26;16:1702408. doi: 10.3389/fmicb.2025.1702408 (PMC12689874; doi:10.3389/fmicb.2025.1702408)
Supplement: Supplementary file 1 [file Data_Sheet_1.docx]

## Supplementary information


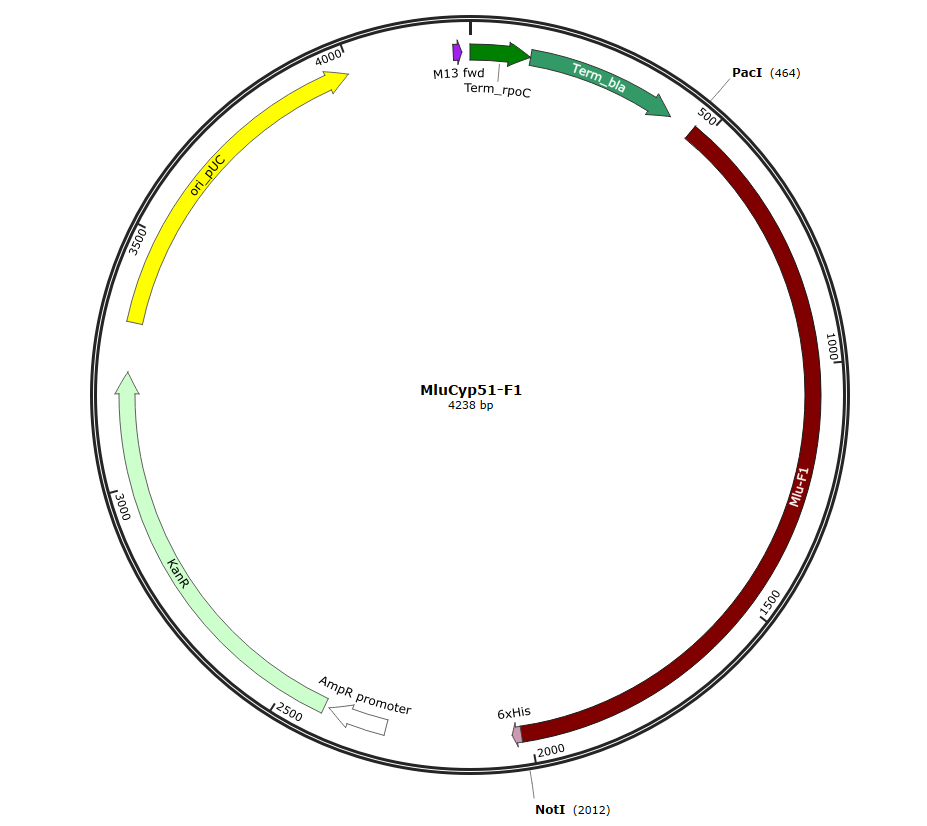


a


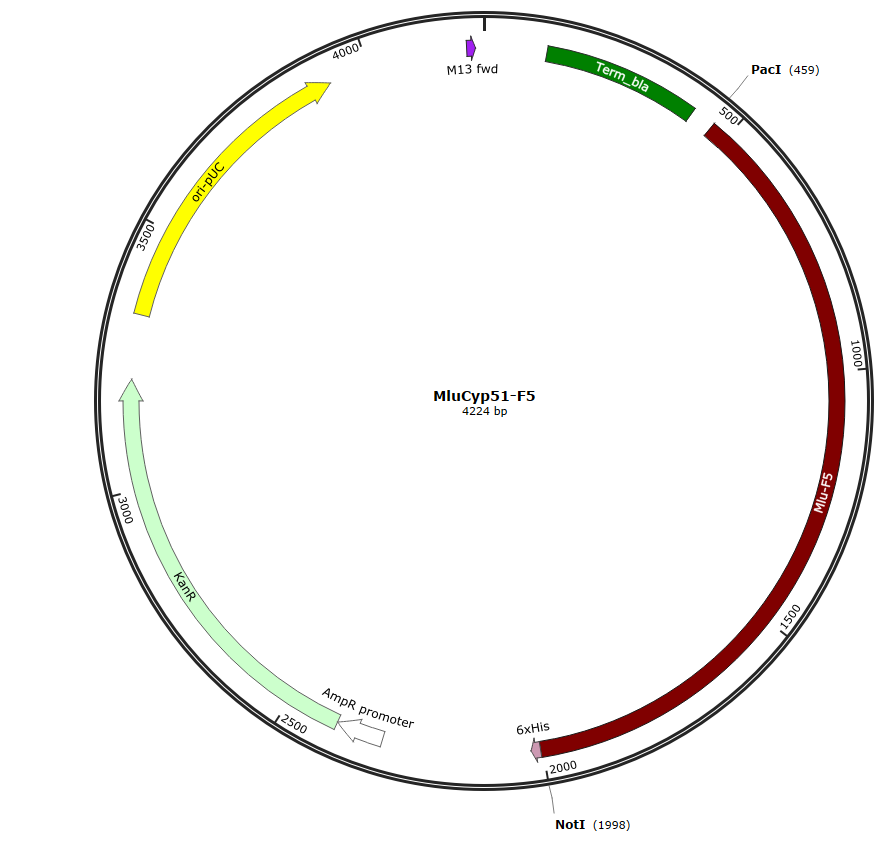


b


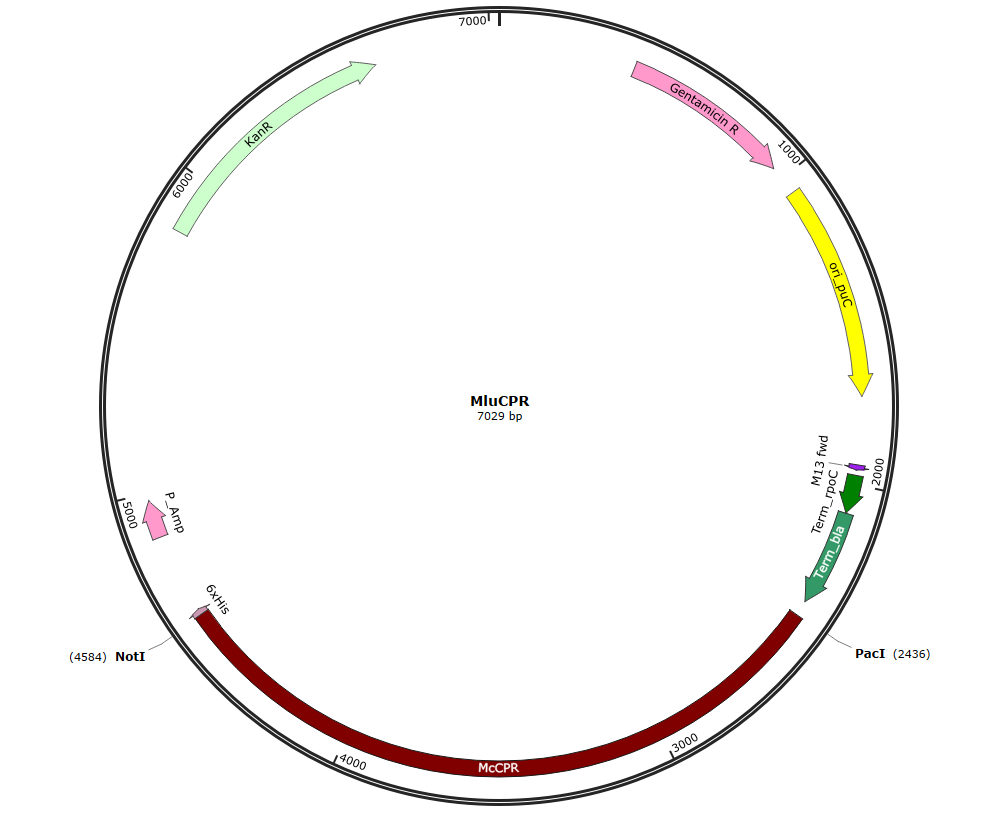


c

**Fig. S 1. Plasmids including codon-optimized heterologous MluCyp51 versions or the cognate P450 reductase (MluCPR) supplied by ATUM (Newark, CA, USA).**

High-copy plasmids with a kanamycin resistance marker, suitable for expression in Escherichia coli, were used (backbone size: 2667-4863 nt). The inserts for (a) MluCyp51-F1, (b) MluCyp51-F5 and (c) MluCPR were 1,594, 1,522 and 2,194 base pairs, respectively. Plasmid maps were created using SnapGene Viewer (Version 5.3.1 GSL Biotech, Boston, MA, USA).

Legend: Mlu: Mucor lusitanicus, 6xHIS: His-Tag, KanR: kanamycin resistance, AmpR: ampicillin resistance, ori_pUC: plasmid backbone, M13 fwd: M13 vector, Term_rpoC: RNA polymerase terminator, R: resistance, NotI and PacI: restriction enzymes.

**Table S 1. Host and recombinant Saccharomyces cerevisiae strains used and created in this study.**

| Strain | Strain name | Genotype | Ref. |
| --- | --- | --- | --- |
| Y1857 | AD∆∆ | MATα PDR1-3 Δ*yor1::hisG* Δ*snq2::hisG* Δ*pdr3::hisG* Δ*pdr10::hisG* Δ*pdr11::hisG* Δ*ycf1::hisG* Δ*pdr5::hisG* Δ*pdr15::hisG* Δ*pdr5::hisG* Δ*pdr15::hisG*, Δ*ura3* Δ*his1::dpl200* | (25) |
| Y2494 | AD∆∆Gal | AD∆∆ ∆*ERG11pro::GAL1pro* | (30) |
| Y2300 | AD∆∆ *PDR5::ScERG11* ∆*ScErg11* | AD∆∆ *PDR5::ScERG11* ∆*ScErg11* | (32) |
| Y941 | AD∆ *ScERG11* | AD∆ *PDR5::ScERG11* | (55) |
| SC2343^(1)^ | MluCPR | AD∆∆Gal *PDR15::MluCPR*-6xHIS*::URA3* | This study |
| SC2344^(1)^ | MluCyp51-F1 | AD∆∆Gal *PDR5::MluCyp51-F1*-6xHIS*::LoxP-HIS1* | This study |
| SC2345^(1)^ | MluCyp51-F5 | AD∆∆Gal *PDR5:: MluCyp51-F5*-6xHIS*::LoxP-HIS1* | This study |
| SC2346^(2)^ | MluCyp51-F1+CPR | AD∆∆Gal *PDR5:: MluCyp51-F1*-6xHIS*::LoxP-HIS1* *PDR15::MluCPR*-6xHIS*::URA3* | This study |
| SC2347^(2)^ | MluCyp51-F5+CPR | AD∆∆Gal *PDR5::* *MluCyp51-F5*-6xHIS ::*LoxP-HIS1* *PDR15::MluCPR*-6xHIS::*URA3* | This study |
| SC2351^(2)^ | MluCyp51-F5 F129Y A293V+CPR | AD∆∆Gal PDR5:: *MluCyp51*-F5-6xHIS, F129Y, A293V-6xHIS*::LoxP-HIS1 PDR15::MluCPR*-6xHIS::*URA3* | This study |
| SC2352^(2)^ | MluCyp51-F5 A293V+CPR | AD∆∆Gal *PDR5:: MluCyp51-F5*-6xHIS*,* A293V*-*6xHIS*::LoxP-HIS1 PDR15::MluCPR*-6xHIS*::URA3* | This study |
| SC2353^(2)^ | MluCyp51-F5 F129Y+CPR | AD∆∆Gal *PDR5:: MluCyp51-F5*-6xHIS*,*  F129Y*-*6xHIS *::LoxP-HIS1 PDR15::MluCPR*-6xHIS::*URA3* | This study |
| Y2653 | RaCyp51-F5 | AD∆∆Gal *PDR5::RaCyp51-F5*-6xHIS*::LoxP-HIS1* | unpublished |
| Y2659 | RaCyp51-F5+CPR | AD∆∆Gal *PDR5::RaCyp51-F5*-6xHIS*::LoxP-HIS1 PDR15::RaCPR*-6xHIS::*URA3* | unpublished |
|  |  |  |  |
|  |  |  |  |

Legend: MATα: mating type alpha; ∆: Deletion, GAL1pro: Gal1 promoter-galactose-inducible; Mlu: Mucor lusitanicus, PDR5: PDR5 locus, PDR15: PDR15 locus, 6xHIS: Tag of 6 histidines; LoxP-HIS1: His selection marker with attached cre/Lox system, URA3: ura selection marker. ^(1)^Parental strain is Y2494, ^(2)^Parental strain is SC2343.

**Table S 2. Oligonucleotides used in this study.**

This table lists all oligonucleotides used in the study, including their sequences, purposes, and relevant annotations.

| **Name** | **Sequence 5'-3'** | **Target** | **Purpose** |
| --- | --- | --- | --- |
| pABC3-PacI-F | CCGCTCGTTCGAAAGACTTAATTAAAAAATG | *PDR5* | Amplification GOI from plasmid, used for MluCyp51-F1 CPR, MluCyp51-F5 CPR and CPR; sequencing Primer |
| Not1-6x His-R | GAATTTAATGATGGTGATGATGGCCGCC | 6x His Tag | Amplification GOI from plasmid, used for MluCyp51-F1 CPR, MluCyp51-F5 CPR and CPR |
| PDR5F | GAACATGAACGTTCCTCAGCGCG | *PDR5* | Amplification US region *PDR5* |
| pABC3-PacI-R | CATTTTTTAATTAAGTCTTTCGAACGAGCGG | *PDR5* | Amplification US region *PDR5*, *PDR15*, colony PCR Integration control *PDR5*, *PDR15* |
| Not1 6x His-F | GGCGGCCGCCATCATCACCATCATCATTAAATTC | 6x His Tag | Amplification DS region *PDR5*, *PDR15* |
| PDR5 288DS-R | CCGTAAGGCACAGTTAAGAAATAATG | *PDR5* | Amplification DS region *PDR5*, creation linear transformation cassette with *PDR5* flanking sites |
| MRP20-697ORF-F | CGAGAGATAGACAATAAGCGAGAG | *PDR15* | Amplification US region *PDR15* |
| PDR15DS-R | GATGGAATAATCCAGTTCGACTCTG | *PDR15* | Amplification linear transformation fragment |
| PDR5-F-v2 | GCAGTCCCTTACATAGTACACAAC | *PDR5* | Creation linear transformation cassette with PDR5 flanking sites, colony PCR integration control *PDR5* |
| PDR15US-F | GTCACGCCGCCGAACTGCAGCGCGC | *PDR15* | Amplification linear transformation fragment |
| MluCyp51-F5 Y129-F | CGTGTTCGGTGACGACATTGTTT**AC**GACGCACCACATTCTGTATTCATG | MluCyp51-F5 | Exchange AA F5 F129Y |
| MluCyp51-F5 Y129-R | CATGAATACAGAATGTGGTGCGTC**GT**AAACAATGTCGTCACCGAACACG | MluCyp51-F5 | Exchange AA F5 F129Y |
| MluCyp51-F5 V291-F | CAGATTTGTGGAATCTTGACTGCAG**T**GTTATTCGGGGGACAACACACATC | MluCyp51-F5 | Exchange F5 AA A293V |
| MluCyp51-F5 V291-R | GATGTGTGTTGTCCCCCGAATAAC**A**CTGCAGTCAAGATTCCACAAATCTG | MluCyp51-F5 | Exchange F5 AA A293V |
| PDR5 US 126-F | TGTGTTAGTTATCACTCGACTTTG | *PDR5* | Sequencing primer |
| PGK1-R | TCGGATAAGAAAGCAACACCTGG | *PGK1* Terminator | Sequencing primer |
| ScHIS1-ORF27-F | ATCGGTTAGATGGTTCACCAAATCC | *HIS1* | Sequencing primer |
| MluF5 637-F | GGCGTTGCACAACTGTACTACG | MluCyp51 | Sequencing primer |
| MluCPR 701-F | GGCCAGCATTCTGTGAAGCGTTAG | MluCPR | Sequencing primer |
| MluCPR ORF 701-F | GGCCAGCATTCTGTGAAGCGTTAG | MluCPR | Sequencing primer |
| ScUra ORF322-R | CCGCAGAGTACTGCAATTTGACTG | *URA3* | Sequencing primer |

Legend: PDR5: PDR5 locus, F: forward, R: reverse, US: upstream, DS: downstream, PacI: PacI cutting site, PDR15: PDR15 locus, v2: version 2, ORF: open reading frame, pABC: plasmid backbone, PGK1: PGK1 terminator, Mlu: Mucor lusitanicus, Not1: Not1 cutting site, GOI: gene of interest, AA: amino acid, Ura: URA3. All oligonucleotides were ordered from metabion International AG, Planegg, Germany.


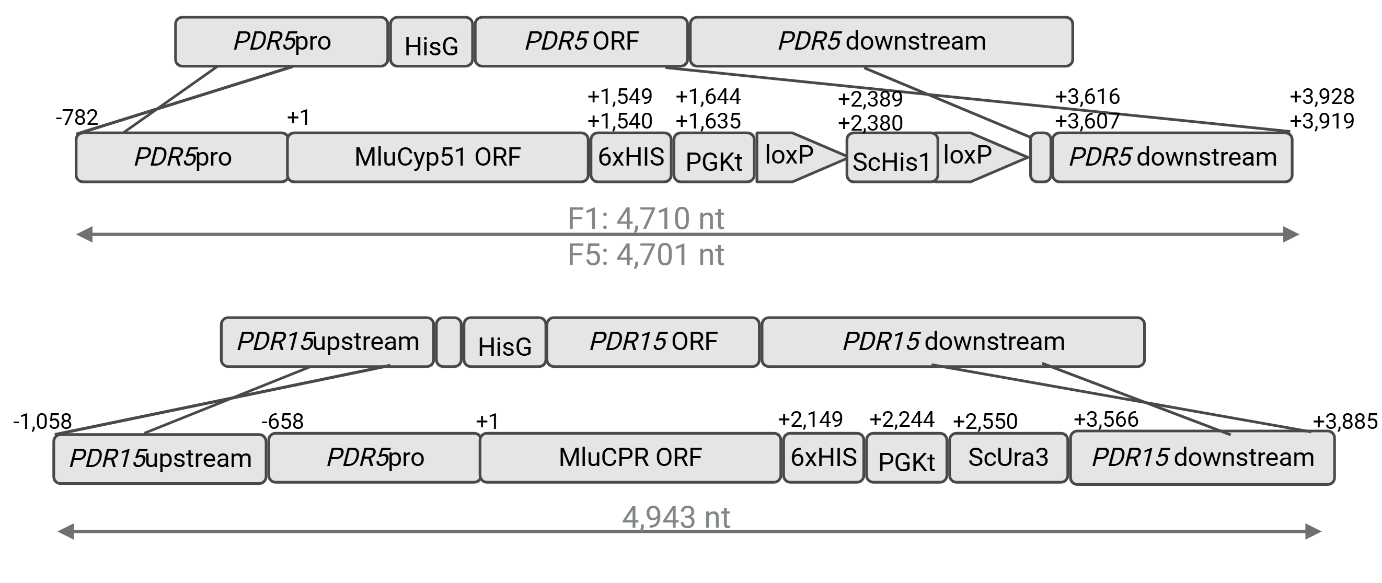


b

a

**Fig. S 2. Transformation strategy for the insertion of heterologous genes into (a) the PDR5 and (b) the PDR15 locus.**

The length of the introduced transformation cassettes are 4,710 nucleotides (nt) for MluCyp51-F1 (upper numbers), 4,701 nt for MluCyp51-F5 (lower numbers) and 4,943 nt for MluCPR. This figure was created with BioRender.com.

Legend: PDR5: PDR5 locus, PDR15: PDR15 locus, ORF: open reading frame, PGKt: PGK1 terminator, Mlu: Mucor lusitanicus, 6xHIS: hexahistidine tag, HIS1: histidine selection marker, URA3: uracil selection marker, Sc: Saccharomyces cerevisiae.

**Fig. S 3. Agarose gel confirming inserts at the PDR5 and PDR15 loci in strains created in the present study.**

DNA fragments were obtained by colony PCR using outer primer pairs. Lanes 1–5 show the amplification of MluCyp51 isoforms at the PDR5 locus using primers PDR5F and PDR5 288DS-R. The strains are presented in the following order: MluCyp51-F1+CPR, MluCyp51-F5+CPR, MluCyp51-F5 F129Y A293V+CPR, MluCyp51-F5 A293V+CPR, and MluCyp51-F5 F129Y+CPR. Lanes 6–10 show the amplification of the cognate reductase at the PDR15 locus using primers PDR15US-F and PDR15DS-R, following the same order of strains as in lanes 1–5.


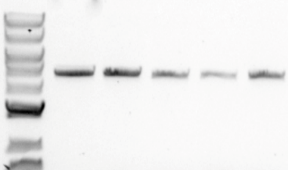

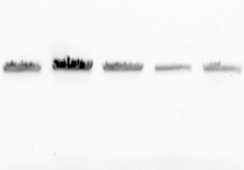


1

2

3

4

5

6

7

8

9

10

10 kb

8 kb

6 kb

5 kb

4 kb

3 kb

2 kb

L

**Figure S3.**

**Table S 3.** **Confirmed sequences obtained by Sanger DNA sequencing**.

Open reading frames (ORFs) of (a) MluCyp51-F1 (strain SC2346), (b) MluCyp51-F5 (strain SC2347; highlighted sequence and underlined ATG represents alternative protein start codons), and (c) MluCPR (strain SC2347) were confirmed from the genomic DNA of the generated strains. The hexahistidine tag and termination sequences are highlighted in bold.

| a | ATGGCAGTGCTTTCAACACTTGTGACTTCCTTACCATCTCTTTCGAATACGGTATTCTACGCTTTCCTGGCCTTGGTTGTTTACATTGTGTTGGATATAGTCAACCAACTATTCGGTCCTAAAGACGCAAAGCAACCACCAGTTGTTTTCTCTTGGATTCCATTTATGGGTAATGCAGTTGAGTTTGGCATTAACCCAATTAAGTTTTTACAAAATTGTCAGAAAAAGTACGGGGACGTCTTTACCTTCAGAATGGTCGGTAAAAAGGTCACAGTATTACTTGGCGCTGACGGTAATCAATTCGTGTTTAACTCCAAGCAAAATCTGAGTTCAGCTGCAGAAGCATACAATGATATGACCAAATATGTTTTCGGAAGAGAAGTCGTCTACGATGCACCTCACTCAGTTTTCCTAGAGCAGAAGCGTTTCATCAAGGCCGGCTTAAACACTGAGTGCTTCAGACAACATGTACCAATGATAGTGGATGAAGTTACAGACTTCTTCAAAGACTTCAAAAAGCCTACCGGAACATTTGATGCATACCATACATTCGGATCATTGATCATCTGTACAGCTTCTAGGTGCCTGATGGGAAAGGAAATTAGAGCAGAGTTGGATGGTTCAGTAGCCCAGCTGTACTACGATTTGGATCAAGGTTTCCAACCAATCAACTTCATTTTTCCTAACTTGCCTCTACCAAGCTATCGAAAGAGAGATGAAGCGTGTAAGAAAATGGCTGAGTTGTACTCTTCAATTATCCAACGTAGAAAGGCAGAGAATGATAACACAAACGCCGATCTTCTTCAAGCTCTAATGGAATCTACATACAAAGATGGTACACCAGTTCCTGACAAGCATATCGCAGGCATGATGATTGCAGTTCTATTTGGTGGACAACATACCAGTGCCACAACTACAGCTTGGACCTTGTTGGAATTGGCTGCCAGACCAGATTTGATTAGAGCTCTCAGAGAGGAACAAATCGAAAAGTTAGGTTCTCTCAAGACCGACTTAACATTCGAAAACCTCAAGGAATTAACTTTGTTGGAAAATTGTGTAAGAGAAACACTAAGATTGCACCCACCAATTTTCCAGATGATGAGAAAGGTCATCGCGGAAAAAGTTACATTTGAAAAGACTGGACACGACATCCCTAAGGGTAACTTTTTGTGCGCGGTACCTGGTGTTACTCAAGTTGATGAGACATACTTTAAAGACCCACTTAAGTACAACCCTATGAGATGGGTTGATTTAGATGATCCAGTGCACAACATGGAAGCCGGGGATGATGCAGATGTAGATTACGGGTTCGGTGCTGTTGGGATTAGTTCTAAAAACCCATTCTTACCTTTTGGTGCTGGCAGACACAGATGTATCGGTGAAACTTTCGGTTACTTACAGATCAAGACTATCATTGCTACAATGATACGTATGTTTGACATAGAGTTAGAAGAGGGTAAATCTGTTCCAAAGCCAGATTACACTTCCATGGTCGTTGTGCCTGAAAAGCCAGCTAACATTAAGTATACTTGGCGTGAA**GGCGGCCGCCATCATCACCATCATCATTAA** |
| --- | --- |
| b | ATGGCTATAATTTCTACCTTGTTATCAGGATTTTCAGTCTCTGAGTTCATTTACAAGGCATTCATGGCAATAGTTTTCTACTGGGGATTCCACATCTTATCTCAATTAGTGATTCCTAAAGACCCAAAAATGGTCCCAACTGTGTTCTCATGGGTTCCAATCATGGGTAACGCGATCGAGTTTGGTAAAGATCCTATCACATTCTTGCAGAACTGCCAGAAAAAGTACGGTGATGTATTCACATTCCAATTGTTGAACAAAAAGGTAACAGCTTGTCTAGGGCCAGATGGCAACCAATTTGTTTTCAACGCCAAACAAAATGTTGCCTCCGCTGCCGCAGCATACAATCATATGACAAAATACGTGTTCGGTGACGACATTGTTTTTGACGCACCACATTCTGTATTCATGGAACAAAAAAGATTTATTAAGGGTGGTTTGAATATCGAAACTTTTCGTAAAGATGTGCCTCTTATTCTAGATGAATGTCACAAGTTCTTCGATAAGTTCGAAAAGACTGGGGTTGTCGATTCTTACAAAATGTTTGGGTCGCTAATCATCAACACGGCCTCCAGGGCTTTGTTAGGTCCAGAAATTAGAAATGCTTTGGATGATGGCGTTGCACAACTGTACTACGACCTAGACCAAGGTTTCAGACCAATTAACTTCATGTTTCCAAACTTACCATTGCCTGCTTACAGAAAGAGAGATGAAGCCAGAGAAAAGTTAGCCGTTCTTTACGAGAAGATTATCGAAAACAGACGTTCAGATAAGTCTGGCACCCAACACGATGACCTTTTACAAGCATTAATGGATGCAAGATACAAGGATGGTTCTGCACTTCCAGATCATCAGATTTGTGGAATCTTGACTGCAGCGTTATTCGGGGGACAACACACATCATCTACAACAAGCGCTTGGACCATATTGGAACTAGCTCACAGACCAGATGTCGTCAAAGCCGTTAGACAGGAAATGATTGAAAAGTTAGGTTCACTTCAAGTCGAGTTCACATATGACAATCTAGAACAGCTCACCTTTTTGGAAGATGTTGTAAGAGAGACTCTCAGACTCCACCCTCCTATTTTCAACATGATGAGAAGAGTGGTTGCAGACAAAATGGTTTATGAAAAGTCAGGCCTGGAAATTCCAAAGGGTAACTTCCTGTGTGCTGCGCCTGGGGTCACACAAATAGATCCAACATACTTTCATAACCCAATGAAATGGGAGCCTAAGCGTTGGTCAGAAAAGACAGATCCTGTTCATCAATTAGAGGTTGGTGATGACGCAAATATTGATTACGGCTTTGGTGCTGTCGGTATTTCTAGTAGAAGTCCATTCTTGCCATTCGGTGCTGGACGACATAGATGCATCGGAGAAAAGTTCGGTTACTTGCAATTGAAGGCTATAATCGGAACATTTATCAAGCGTTTCGATTTTGAGGGACTTACCGAAAATGTGCCAAAGCCTGATTATACTTCTATGGTAGTTGTACCAGAGGATTCCAGAATAAGATTCACTAGACGTGAC**GGCGGCCGCCATCATCACCATCATCATTAA** |
| c | ATGACCAGATCTTCACAACATTTACTAGATTCATTGGATATCATCTTCTTGGGAACAATTGGGCTAGGTACAATTGCATGGTTCGCA**A**GACACCAAATCGCTGACAAGCTCTTTAAGTCATCTAAGCCAGAAATCAAGCCAGCTGCCGATACTAAAACTGGGCCACCTAAAAAAGAGAGAAACTTCGTAAAGGTTATGCAACAACAGGGCCGTAGAGTCATTTTCTTCTACGGTTCACAAACTGGGACAGCCGAGGATTATGCTTCAAGATTAGCTAAGGAATGTTCACAGAAATACGGTGTTTCTTCCATGGCAGCAGACATTGAACAATACGACATGTCTTACTTAGATACTGTTCCTGAAGATTTCCTTGTTTTCTTTGTGATGGCTACATACGGTGAGGGTGAACCAACAGACAATGCTGTCGATTTTTGGGATTTGGTTTCAGACGAACAACCTCAATTTTCAGAAGCCGAAGATGAAGAGGCGCCACTTAAAAATTTAAGATACGTTGCATTTGGTTTAGGGAACAAGACCTACGAGCATTACAATGAAGTAATTAGAAACATCGACAAAAGATTGACATGCATGGGTGCTAAGAGAATCGGTGAGAGGGGTGAGGGAGATGATGATGGCTCTTTGGAAGAGGACTTCTTGGCATGGCAGGAAGAGATGTGGCCAGCATTCTGTGAAGCGTTAGGTGTGGATGAGAATAGTGCTTCATCTGGTCCTAGACAAGCTAGTTTTGCAGTCGAGGAACTCTCAGAATTTGATAAGGCCAAGATTTACGTCGGTGAATTGTCTGAATGGTTGAAGGAAGGTGCAAGAGTGGTATACGACGCCAAAAGACCATATAATGCTCCAATAACCTCTACAGATTTGTTCAAGGGCGGGGATAGACATTGTTTACATATGGAAATCGATATTTCTGATTCCAACCTAACATATCAAACCGGCGATCACGTAGCAATATGGCCAACTAACAACGAAATCGAAATAGAAAGACTCGCCAAGGTCCTAGGTTTGTCTGATAAGTTAGACACAGTTATCCG**T**GTTCAAGCACTAGATGCTGCCGCATCTAAGCAATTCCCATTCCCTGTGCCAACTACCTATAGAGCAATCTTCCG**T**CACTACTTGGACATTTGCGCTGCTGTCTCTAGACAGACATTGATGAGCCTGATTGAATACGCTCCTACTGAAAAGTCCAAAGATATCCTTAGAAAGTTAGCCACCGATAAAGACGAATATAGAGTAAGAGTGGGTGATGTGACCAGAAACTTAGGTGAAGTCCTTGAAATGTTAGCTGAGTCTGAAAGTCTTGACGTAGAGGGATCATTCTCTAGTGTACCATTCGATTTGATTGTAGAATCAGTGTCAAGACTTCAACCTAGATACTACTCTATCAGTTCCAGTTCTAAAGAGTCGCCAAAAAAGATTACAGTCACAGTCGTTACTTTGCAATACACGCCAGAAACAACTTCCCCTAGAACAGTTTACGGTGTTAACACAAATTACCTTTGGAGACTGCACGAGTCCATAAACGGCATCGAACCAGATGCTTCATTCCCACATTACTCTATCACCGGACCACGTAACTGTTTATACGACGCAGAATCGAAAGTTGCGAGAGTTCCAGTGCACGTCCG**T**AGATCTCAATTCAAGTTGCCACGTAATCCAACTGTCCCTGTGATTATGGTTGGACCTGGTACAGGTGTCGCACCATTTCGTGGTTTTGTTAGAGAACGTGCGCTTCAGAAAAAAGAGGGAAAGCCAATTGGACCAACTATTCTATACTTTGGATGTAGAAACTCTGCAGAGGATTTCTTGTACGAAGAGGAATGGCCTGAATTATTCGAAACTCTAGGCGAATCCAGCAGAATTATAACGGCATTCAGCAGAGAGACAGCACAAAAGGTTTATGTTCAGCACAGATTAATGGAAAACGGTCCAGAGATGTGGGATCTCCTTGAGAAAGGCGCCTACGTTTACGTTTGCGGCGATGCCAAGGTTATGGCCAGAGATGTTAACCAAACATTCGTAAGATTTGCTCAACAGTTTGGTGGGCTGGAAGAGGAAAAGGCTCAAGATTACGTCAAAAATCTGAGAAACACAGGTAGATACCAAGAGGACGTTTGGTCC**GGCGGCCGCCATCATCACCATCATCATTAA** |

**Table S 4. Primary amino acid sequence of inserted heterologous genes**.

Sequence coverage was obtained by mass spectrometry of trypsin-digested fragments from (a) MluCyp51-F1, (b) MluCyp51-F5 and (c) MluCPR. The identified amino acids sequences are highlighted in grey. An overview of the strains and inserts is provided in Table S 1. Fragments containing MluCyp51-F1 Y130 and MluCyp51-F5 Y129 that were detected are highlighted in yellow, while MluCyp51-F1 V294 and MluCyp51-F5 A293 that were not detected are highlighted in red.

|  | **a Coverage primary sequence 60.54%,** obtained from strain SC2346 | | |
| --- | --- | --- | --- |
|  |  | 1 | MAVLSTLVTS LPSLSNTVFY AFLALVVYIV LDIVNQLFGP KDAKQPPVVF |
|  |  | 51 | SWIPFMGNAV EFGINPIKFL QNCQKKYGDV FTFRMVGKKV TVLLGADGNQ |
|  |  | 101 | FVFNSKQNLS SAAEAYNDMT KYVFGREVVY DAPHSVFLEQ KRFIKAGLNT |
|  |  | 151 | ECFRQHVPMI VDEVTDFFKD FKKPTGTFDA YHTFGSLIIC TASRCLMGKE |
|  |  | 201 | IRAELDGSVA QLYYDLDQGF QPINFIFPNL PLPSYRKRDE ACKKMAELYS |
|  |  | 251 | SIIQRRKAEN DNTNADLLQA LMESTYKDGT PVPDKHIAGM MIAVLFGGQH |
|  |  | 301 | TSATTTAWTL LELAARPDLI RALREEQIEK LGSLKTDLTF ENLKELTLLE |
|  |  | 351 | NCVRETLRLH PPIFQMMRKV IAEKVTFEKT GHDIPKGNFL CAVPGVTQVD |
|  |  | 401 | ETYFKDPLKY NPMRWVDLDD PVHNMEAGDD ADVDYGFGAV GISSKNPFLP |
|  |  | 451 | FGAGRHRCIG ETFGYLQIKT IIATMIRMFD IELEEGKSVP KPDYTSMVVV |
|  |  | 501 | PEKPANIKYT WREGGRHHHH HH |
|  |  |  |  |
|  | **b Coverage primary sequence 69.28%,** obtained from strain SC2347 | | |
|  |  | 1 | MAIISTLLSG FSVSEFIYKA FMAIVFYWGF HILSQLVIPK DPKMVPTVFS |
|  |  | 51 | WVPIMGNAIE FGKDPITFLQ NCQKKYGDVF TFQLLNKKVT ACLGPDGNQF |
|  |  | 101 | VFNAKQNVAS AAAAYNHMTK YVFGDDIVFD APHSVFMEQK RFIKGGLNIE |
|  |  | 151 | TFRKDVPLIL DECHKFFDKF EKTGVVDSYK MFGSLIINTA SRALLGPEIR |
|  |  | 201 | NALDDGVAQL YYDLDQGFRP INFMFPNLPL PAYRKRDEAR EKLAVLYEKI |
|  |  | 251 | IENRRSDKSG TQHDDLLQAL MDARYKDGSA LPDHQICGIL TAALFGGQHT |
|  |  | 301 | SSTTSAWTIL ELAHRPDVVK AVRQEMIEKL GSLQVEFTYD NLEQLTFLED |
|  |  | 351 | VVRETLRLHP PIFNMMRRVV ADKMVYEKSG LEIPKGNFCZ AAPGVTQIDP |
|  |  | 401 | TYFHNPMKWE PKRWSEKTDP VHQLEVGDDA NIDYGFGAVG ISSRSPFLPF |
|  |  | 451 | GAGRHRCIGE KFGYLQLKAI IGTFIKRFDF EGLTENVPKP DYTSMVVVPE |
|  |  | 501 | DSRIRFTRRD GGRHHHHHH |
|  |  |  |  |
|  | **c Coverage primary sequence 62.33%,** obtained from strain SC2347 | | |
|  |  | 1 | MTRSSQHLLD SLDIIFLGTI GLGTIAWFAR HQIADKLFKS SKPEIKPAAD |
|  |  | 51 | TKTGPPKKER NFVKVMQQQG RRVIFFYGSQ TGTAEDYASR LAKECSQKYG |
|  |  | 101 | VSSMAADIEQ YDMSYLDTVP EDFLVFFVMA TYGEGEPTDN AVDFWDLVSD |
|  |  | 151 | EQPQFSEAED EEAPLKNLRY VAFGLGNKTY EHYNEVIRNI DKRLTCMGAK |
|  |  | 201 | RIGERGEGDD DGSLEEDFLA WQEEMWPAFC EALGVDENSA SSGPRQASFA |
|  |  | 251 | VEELSEFDKA KIYVGELSEW LKEGARVVYD AKRPYNAPIT STDLFKGGDR |
|  |  | 301 | HCLHMEIDIS DSNLTYQTGD HVAIWPTNNE IEIERLAKVL GLSDKLDTVI |
|  |  | 351 | RVQALDAAAS KQFPFPVPTT YRAIFRHYLD ICAAVSRQTL MSLIEYAPTE |
|  |  | 401 | KSKDILRKLA TDKDEYRVRV GDVTRNLGEV LEMLAESESL DVEGSFSSVP |
|  |  | 451 | FDLIVESVSR LQPRYYSISS SSKESPKKIT VTVVTLQYTP ETTSPRTVYG |
|  |  | 501 | VNTNYLWRLH ESINGIEPDA SFPHYSITGP RNCLYDAESK VARVPVHVRR |
|  |  | 551 | SQFKLPRNPT VPVIMVGPGT GVAPFRGFVR ERALQKKEGK PIGPTILYFG |
|  |  | 601 | CRNSAEDFLY EEEWPELFET LGESSRIITA FSRETAQKVY VQHRLMENGP |
|  |  | 651 | EMWDLLEKGA YVYVCGDAKV MARDVNQTFV RFAQQFGGLE EEKAQDYVKN |
|  |  | 701 | LRNTGRYQED VWSGGRHHHH HH |

**Table S 5**. **List of 10 most abundant proteins found in crude membrane preparations**.

Proteins from strains expressing MluCyp51-F1, MluCyp51-F5 and MluCPR were identified by mass spectrometry. The heterologous proteins of M. lusitanicus are highlighted in grey, even if they are not the most abundant.

| **ID** | **Gene Name** | **Description** | **Abundance** |
| --- | --- | --- | --- |
| MluCyp51-F1 | Cyp51-F1 | Sterol 14α-demethylase | 3,20E+09 |
| YDL229W | SSB1 | Stress-seventy subfamily B | 8,79E+10 |
| YAL005C | SSA1 | Stress-seventy subfamily B | 4,85E+10 |
| YJR045C | SSC1 | Stress-seventy subfamily B | 2,20E+10 |
| YER165W | PAB1 | Poly(a)-binding protein | 1,51E+10 |
| YCL043C | PDI1 | Protein disulphide-isomerase | 1,23E+10 |
| YBR118W | TEF2 | Translation elongation factor | 1,22E+10 |
| YOR204W | DED1 | Defines essential domain | 1,17E+10 |
| YHR042W | NCP1 | NADP cytochrome P450 reductase | 1,04E+10 |
| YNL121C | TOM70 | Translocase of the outer mitochondrial membrane | 8,85E+09 |
| MluCyp51-F5 | Cyp51-F5 | Sterol 14α-demethylase | 2,45E+11 |
| YML120C | NDI1 | NADH dehydrogenase internal | 3,35E+10 |
| YFL018C | LPD1 | Lipoamide dehydrogenase | 3,10E+10 |
| YBR118W | TEF2 | Translation elongation factor 2 | 2,64E+10 |
| YMR145C | NDE1 | NADH dehydrogenase, external | 1,86E+10 |
| YLR044C | PDC1 | Pyruvate decarboxylase | 1,35E+10 |
| YNL071W | LAT1 | Dihydrolipoamide acetyltransferase component | 1,27E+10 |
| YOR374W | ALD4 | Aldehyde dehydrogenase | 1,10E+10 |
| YKR016W | FCJ1 | Mitochondrial contact site and cristae organizing system | 9,19E+09 |
| YOR198C | BFR1 | Brefeldin A resistance | 8,07E+09 |
| CPR | CPR | Cytochrome P450 reductase | 1,52E+11 |
| YMR186W | HSC82 | Cytoplasmic chaperone of the Hsp90 family | 1,81E+10 |
| YBR118W | TEF2 | [Translation elongation factor](https://www.yeastgenome.org/locus/S000000322#reference) | 8,62E+09 |
| YKR009C | FOX3 | [Fatty acid oxidation 3](https://www.yeastgenome.org/locus/S000001717#reference) | 7,57E+09 |
| YOR361C | PRT1 | Subunit of the eukaryotic translation initiation factor | 3,70E+09 |
| YOR165W | SEY1 | Synthetic enhancement of YOP1 | 3,61E+09 |
| YLR429W | CRN1 | Coronin; cortical actin cytoskeletal component | 3,10E+09 |
| YMR089C | YTA12 | [Yeast TAT-binding analog](https://www.yeastgenome.org/locus/S000004695#reference) | 2,51E+09 |
| YNL061W | NOP2 | Nucleolar protein | 2,21E+09 |
| YPL240C | HSP82 | Heat shock protein | 1,71E+09 |


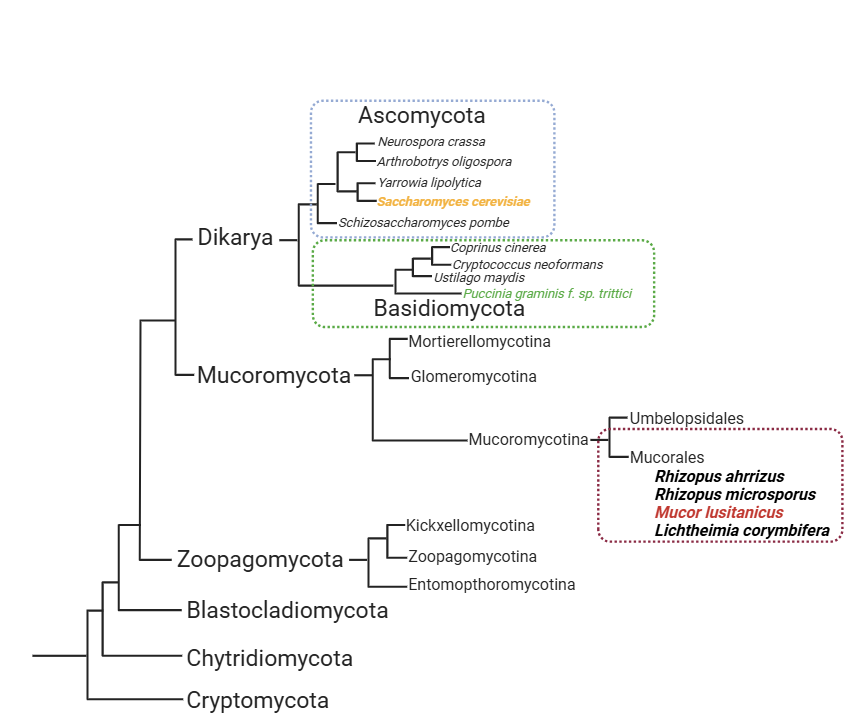


617 million years ago

**Fig. S 4. Schematic illustrating the phylogenetic relationships of Mucorales to Basidiomycota and Ascomycota.** The target organism M. lusitanicus belongs to the Mucoromycotina, a sister group of the Dikarya, which comprises Basidiomycota and Ascomycota (including the *Saccharomyces cerevisiae* host). Adapted from (1, 5, 36). Created with BioRender.com.

**Fig. S 5. Protein expression of MluCyp51-F1 and MluCyp51-F5 from the PDR5 locus without the cognate reductase.** (a) Calculated relative expression in recombinant MluCyp51 constructs expressed from the PDR5 locus (b) Western Blot analysis and (c) SDS-PAGE of the constructed strains. Strains are shown as follows: Lane 1: Y2300, Lane 2: AD∆∆, Lane 3: MluCyp51-F1, Lane 4: MluCyp51-F5, Lane L: Protein broad range Standard. The asterisk (*) indicates the recombinant Cyp51, the arrow indicates a tubulin-like protein (49 kDa). An overview of the strains and inserts is provided in Table S 1.

**Fig. S 6. Relative MluCPR expression of strains MluCYP51-F1+CPR, MluCyp51-F5+CPR and MluCYP51-F5+CPR variants.** An overview of the strains and inserts is provided in Table S 1.


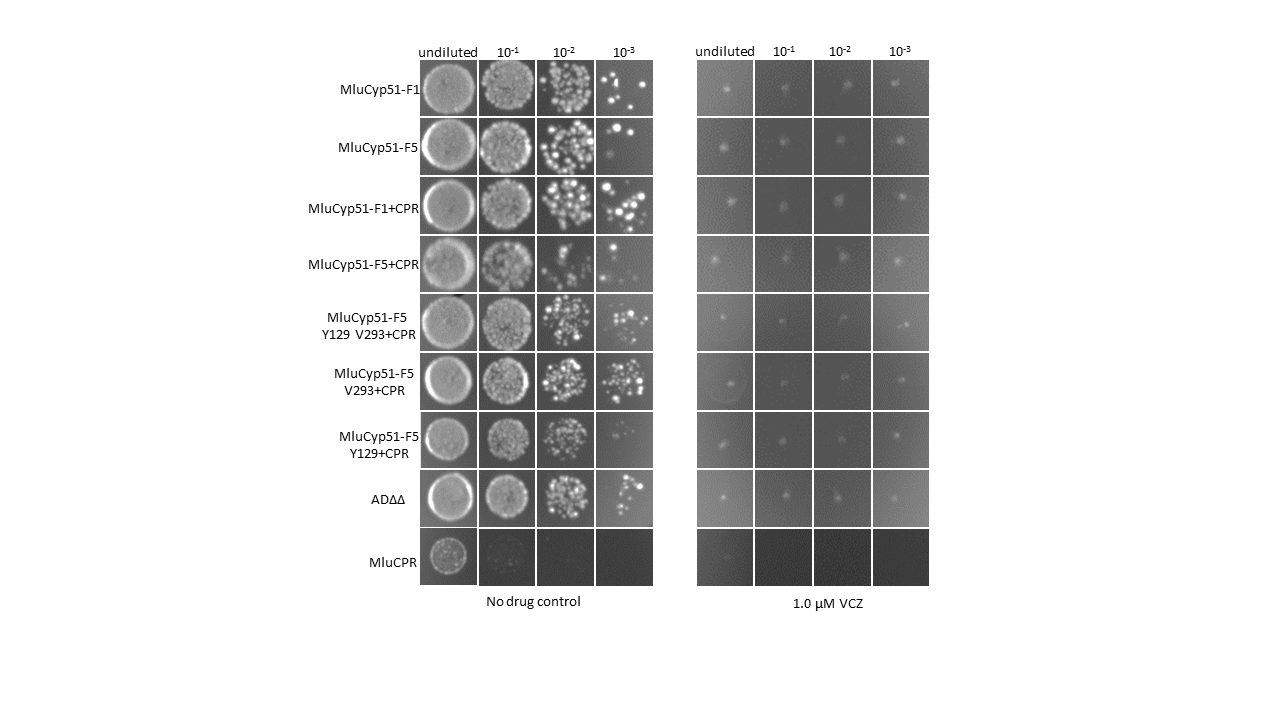


**Fig. S 7. Spot susceptibility assay of all strains under no drug conditions and in the presence of 1.0 µM PCZ.**

**Table S 6. Mean minimal inhibitory concentration (MIC) values (mg/L) for all strains and antifungals tested.**

| Strains | AMB | FCZ | VCZ | IVZ | ITZ | PCZ |
| --- | --- | --- | --- | --- | --- | --- |
| AD∆∆ | 1.0 ± 0.0 | 1.0 ± 0 | 0.02 ± 0.0 | 0.02 ± 0.0 | 0.05 ± 0.0 | 0.03 ± 0.0 |
| MluCPR | 2.0 ± 0.0 | 0.7 ± 0.3 | 0.01 ± 0.0 | 0.01 ± 0.0 | 0.04 ± 0.02 | 0.03 ± 0.0 |
| MluCyp51-F1 | 1.0 ± 0.0 | 1.3 ± 0.6 | 0.02 ± 0.01 | 0.02 ± 0.01 | 0.01 ± 0.0 | 0.01 ± 0.0 |
| MluCyp51-F5 | 0.7 ± 0.3 | **42.7 ± 18.5** | **0.50 ± 0.43** | 0.07 ± 0.05 | 0.04 ± 0.02 | 0.01 ± 0.0 |
| MluCyp51-F1+CPR | 1.7 ± 0.6 | 10.7 ± 4.6 | 0.17 ± 0.07 | **0.17 ± 0.07** | 0.04 ± 0.02 | 0.01 ± 0.0 |
| MluCyp51-F5+CPR | 2.7 ± 1.2 | **256 ± 0.0** | **4.0 ± 0.0** | **4.0 ± 0.0** | **0.21 ± 0.07** | 0.05 ± 0.02 |
| MluCyp51-F5 F129Y+CPR | **3.0 ± 1.7** | 4.0 ± 0.0 | 0.25 ± 0.0 | 0.06 ± 0.0 | 0.06 ± 0.0 | 0.06 ± 0.0 |
| MluCyp51-F5 A293V+CPR | 1.5 ± 0.9 | 16.0 ± 0.0 | **0.50 ± 0.0** | **0.13 ± 0.0** | **0.13 ± 0.0** | **0.08 ± 0.04** |
| MluCyp51-F5 F129Y A293V+CPR | 0.5 ± 0.0 | 1.0 ± 0.0 | 0.06 ± 0.0 | 0.02 ± 0.0 | 0.03 ± 0.0 | 0.03 ± 0.0 |

Statistical analysis was performed using one-way ANOVA with Dunnett’s post-hoc test, comparing results to the host strain AD∆∆. Significant differences (p <0.05, n=3) relative to AD∆∆ are highlighted in bold. An overview of the strains and inserts is provided in S 1 Table.

**Fig. S 8. Resistance profiles of strains expressing MluCyp51 variants with or without MluCPR.**

Minimal inhibitory concentrations (MICs) were determined for (a) AMB and (b-f) the azoles. Statistical analysis was performed using one-way ANOVA with Dunnett’s post-hoc test, comparing results to the host strain AD∆∆. Significance levels according to Dunnett’s post-hoc test are indicated as follows: *p-value = 0.0332, **p-value = 0.0021, ***p-value = 0.0002, ****p-value < 0.0001. Data represent n=3 biological replicates. An overview of the strains and inserts is provided in Table S1.

**Fig. S 9. Fold change in mean susceptibility of strains expressing MluCyp51 isoforms compared to the S. cerevisiae host strain AD∆∆.** An overview of the strains and inserts is provided in Table S 1.

**Table S 7. Comparison of mean doubling time (Td, min) under no-drug conditions or with antifungal treatment.**

|  | AD∆∆ | MluCyp51-F1+CPR | MluCyp51-F5+CPR | MluCyp51-F5 F129Y+CPR | MluCyp51-F5 A293V+CPR | MluCyp51-F5 F129Y A293V+CPR |
| --- | --- | --- | --- | --- | --- | --- |
| NDC | 167 | 173 | 163 | 160 | 232 | 171 |
| PCZ 0.1 µM | 365 (2.17) | 1643 (9.49) | 374 (2.29) | 1690 (10.6) | 1887 (8.13) | 8822 (51.6) |
| IVZ 0.1 µM | 189 (1.14) | 183 (1.06) | 177 (1.09) | 202 (1.26) | 182 (0.78) | 1163 (6.80) |
| IVZ 1.0 µM | 241 (1.44) | 204 (1.18) | 162 (0.99) | 180 (1.13) | 263 (1.33) | 1173 (6.86) |
| VCZ 0.1 µM | 157 (0.94) | 193 (1.12) | 165 (1.01) | 162 (1.01) | 247 (1.06) | 926 (5.42) |
| VCZ 1.0 µM | 335 (2.00) | 618 (3.57) | 162 (0.99) | 373 (2.33) | 317 (1.36) | 983 (5.74) |

Strains were challenged with VCZ (0.1µM ≙ 0.035 mg/L and 1 µM ≙ 0.35 mg/L), IVZ (0.1µM ≙ 0.043 mg/L; 1.0µM ≙ 0.43 mg/L) and PCZ; (0.1µM ≙ 0.07 mg/L). Fold changes in doubling time (Td) under treatment compared to NDC are shown in parentheses.

**Fig. S 10. Growth characteristics of strains expressing MluCyp51-F1+CPR and MluCyp51-F5+CPR compared to the S. cerevisiae host in (a) low-dose VCZ and (b) low-dose IVZ.**

Statistical analysis was performed using One-way ANOVA with Dunnett’s post-hoc test. p-values: ns – not significant. Data represent n=3 biological replicates.

**Fig. S 11. Growth curves of recombinant strains compared to the S. cerevisiae host.**

Strains harboring single or combined amino acid substitutions in MluCyp51-F5+CPR (F129Y, A293V, and the combined F129Y A293V) were analyzed. Media contained (a) no drug, (d) 0.1 µM PCZ, (b, e) 0.1 µM and 1.0 µM VCZ, and (c, f) 0.1 µM and 1.0 µM IVZ. Statistical analysis was performed using one-way ANOVA with Dunnett’s post-hoc test. Significance levels according to Dunnett’s post-hoc test are indicated as follows: *p-value = 0.0332, **p-value = 0.0021, ***p-value = 0.0002, ****p-value < 0.0001. Data represent n=3 biological replicates. An overview of the strains and inserts is provided in Table S 1.

**Table S 8.** **Detected percentages of lanosterol, abnormal diols, and ergosterol in all strains with and without azole exposure. Absolute quantities (mean µg/mg fungal dry weight) are displayed in brackets.**

|  |  | **AD∆∆** | **MluCyp51-F1** | **MluCyp51-F5** | **MluCyp51-F1+CPR** | **MluCyp51-F5+CPR** | **MluCyp51-F5 F129Y A293V+CPR** | **MluCyp51-F5 F129Y+CPR** | **MluCyp51-F5 A293V+CPR** |
| --- | --- | --- | --- | --- | --- | --- | --- | --- | --- |
| **NDC** | Lanosterol | 7.4 ± 3.4  (0.37) | 25.9 ± 6.0  (1.15) | 23.1 ± 9.3  (1.13) | 14.7±1.1  (0.30) | 7.9 ± 4.9  (0.17) | 17.1 ± 1.6  (0.20) | 12.8 ± 1.5  (0.11) | 21.9 ± 1.5  (0.32) |
|  | Ergosterol intermediates with a methyl group at C14 | 0.0 ± 0.0  (0.0) | 1.9 ± 0.6  (0.08) | 1.4 ± 0.5  (0.07) | 0.1±0.0  (0.0) | 0.0 ± 0.0  (0.0) | ND | ND | 1.9 ± 0.1  (0.03) |
|  | 14-Methylergosta-8,24(28)-dienol | 0.1 ± 0.1  (0.0) | 3.6 ± 3.6  (0.15) | 2.9 ± 1.3  (0.14) | 1.0±0.2  (0.02) | 0.2 ± 0.3  (0.0) | 0.4 ± 0.3  (0.01) | 0.1 ± 0.1  (0.00) | 0.5 ± 0.0  (0.01) |
|  | 14-Methylergosta-8,24(28)-dien-3,6-diol | 0.0 ± 0.0  (0.0) | 28.8 ± 1.1  (1.24) | 25.1 ± 3.3  (1.11) | 2.4±0.3  (0.05) | 0.0 ± 0.0  (0.0) | 1.7 ± 0.1  (0.02) | 0.1 ± 0.1  (0.01) | 3.2 ± 0.2  (0.05) |
|  | 14-Methylergost-8-en-3,6-diol | 0.0 ± 0.0  (0.0) | 4.2 ± 0.9  (0.20) | 1.0 ± 1.3  (0.03) | 0.0±0.0  (0.0) | 0.0 ± 0.0  (0.0) | 1.2 ± 0.4  (0.02) | 0.5 ± 0.2  (0.00) | ND |
|  | Ergosterol | 84.3 ± 4.9  (1.28) | 36.2 ± 7.0  (1.52) | 46.2 ± 10.8  (1.92) | 79.1 ± 1.3  (1.62) | 87.5 ± 5.3  (1.86) | 77.5 ± 6.4  (0.84) | 83.9 ± 3.4  (0.65) | 70.4 ± 1.6  (1.02) |
| **VCZ 0.1 µM** | Lanosterol | 30.7 ± 6.2  (0.63) | 26.2 ± 3.7  (1.35) | 24.3 ± 7.9  (095) | 18.8±1.2  (0.40) | 8.5 ± 4.3  (0.16) | 25.7 ± 4.5  (0.32) | 19.4 ± 1.3  (0.18) | 23.6 ± 0.8  (0.34) |
|  | Ergosterol intermediates with a methyl group at C14 | 1.0 ± 0.3  (0.02) | 3.6 ± 1.2  (0.19) | 1.7 ± 0.6  (0.06) | 0.2±0.0  (0.0) | 0.0 ± 0.0  (0.0) | ND | ND | 2.1 ± 0.1  (0.03) |
|  | 14-Methylergosta-8,24(28)-dienol | 3.6 ± 1.0  (0.08) | 4.7 ± 0.6  (0.24) | 3.1 ± 1.3  (0.12) | 1.4±0.2  (0.03) | 0.2 ± 0.4  (0.0) | 0.8 ± 0.4  (0.01) | 0.2 ± 0.2  (0.00) | 0.6 ± 0.0  (0.01) |
|  | 14-Methylergosta-8,24(28)-dien-3,6-diol | 4.4 ± 1.5  (0.10) | 42.8 ± 3.3  (2.13) | 27.5 ± 1.6  (1.05) | 4.0±0.6  (0.08) | 0.0 ± 0.0  (0.0) | 3.2 ± 1.2  (0.03) | 0.4 ± 0.1  (0.01) | 3.9 ± 0.2  (0.06) |
|  | 14-Methylergost-8-en-3,6-diol | 0.0 ± 0.0  (0.0) | 3.6 ± 1.3  (0.20) | 2.2 ± 0.9  (0.09) | 0.0±0.0  (0.0) | 0.0 ± 0.0  (0.0) | 1.8 ± 0.8  (0.46) | 1.0 ± 0.4  (0.00) | ND |
|  | Ergosterol | 59.0 ± 8.6  (0.98) | 18.9 ± 6.6  (0.90) | 41.2 ± 9.5  (1.55) | 73.0 ± 0.9  (1.54) | 84.2 ± 4.7  (1.45) | 67.1 ± 7.3  (0.73) | 77.5 ± 3.4  (0.64) | 67.8 ± 1.0  (0.98) |

Table S8 continued.

| **VCZ 1.0 µM** | Lanosterol | 31.0 ± 6.2  (0.65) | 26.3 ± 2.6  (1.42) | 25.7 ± 5.0  (1.24) | 25.5±1.1  (0.69) | 12.9 ± 6.1  (0.27) | 37.4 ± 6.1  (0.58) | 31.5 ± 5.3  (0.31) | 26.6 ± 1.3  (0.36) |
| --- | --- | --- | --- | --- | --- | --- | --- | --- | --- |
|  | Ergosterol intermediates with a methyl group at C14 | 1.1 ± 0.5  (0.03) | 4.0 ± 0.5  (0.22) | 3.1 ± 1.1  (0.15) | 0.7±0.1  (0.02) | 0.0 ± 0.0  (0.0) | ND | ND | 3.0 ± 0.1  (0.04) |
|  | 14-Methylergosta-8,24(28)-dienol | 3.7 ± 1.0  (0.08) | 4.9 ± 0.6  (0.26) | 4.2 ± 0.9  (0.21) | 3.1±0.5  (0.08) | 0.6 ± 0.5  (0.01) | 1.6 ± 0.7  (0.03) | 0.9 ± 0.6  (0.01) | 1.0 ± 0.0  (0.01) |
|  | 14-Methylergosta-8,24(28)-dien-3,6-diol | 4.9 ± 1.6  (0.11) | 43.5 ± 6.5  (2.32) | 37.8 ± 3.8  (1.75) | 13.7±1.4  (0.37) | 0.6 ± 0.5  (0.01) | 8.2 ± 5.0  (0.06) | 2.1 ± 1.0  (0.03) | 7.2 ± 0.2  (0.10) |
|  | 14-Methylergost-8-en-3,6-diol | 0.0 ± 0.0  (0.0) | 5.5 ± 2.9  (0.31) | 1.9 ± 2.6  (0.11) | 0.0±0.0  (0.00) | 0.0 ± 0.0  (0.00) | 3.5 ± 1.5  (0.15) | 2.5 ± 1.1  (0.02) | ND |
|  | Ergosterol | 57.9 ± 9.0  (0.98) | 16.1 ± 3.3  (0.86) | 26.6 ± 5.2  (1.20) | 55.1 ± 2.1  (1.49) | 82.4 ± 6.2  (1.63) | 48.8 ± 13.3  (0.60) | 62.5 ± 8.4  (0.48) | 60.8 ± 1.3  (0.81) |
| **PCZ 0.1 µM** | Lanosterol | 31.1 ± 5.8  (0.74) | 26.4 ± 1.9  (1.37) | 27.9 ± 4.0  (1.47) | 26.9±1.0  (0.81) | 30.7 ± 3.8  (0.80) | 44.2 ± 9.6  (0.98) | 39.1 ± 8.8  (0.50) | 30.5 ± 0.2  (0.61) |
|  | Ergosterol intermediates with a methyl group at C14 | 1.1 ± 0.5  (0.03) | 3.9 ± 0.3  (0.20) | 4.2 ± 0.5  (0.22) | 1.6±0.2  (0.05) | 1.4 ± 0.8  (0.04) | ND | ND | 5.8 ± 0.1  (0.12) |
|  | 14-Methylergosta-8,24(28)-dienol | 3.7 ± 1.1  (0.09) | 4.7 ± 0.5  (0.24) | 5.1 ± 0.8  (0.27) | 4.5±0.4  (0.13) | 4.3 ± 1.7  (0.12) | 2.1 ± 1.0  (0.05) | 1.3 ± 1.2  (0.03) | 2.8 ± 0.0  (0.06) |
|  | 14-Methylergosta-8,24(28)-dien-3,6-diol | 4.9 ± 1.8  (0.13) | 46.6 ± 4.3  (2.37) | 46.7 ± 2.1  (2.44) | 26.9±0.7  (0.80) | 23.4 ± 7.0  (0.64) | 14.7 ± 9.9  (0.15) | 5.5 ± 6.3  (0.07) | 34.0 ± 2.9  (0.69) |
|  | 14-Methylergost-8-en-3,6-diol | 0.0 ± 0.0  (0.00) | 6.3 ± 2.2  (0.39) | 1.3 ± 1.9  (0.07) | 0.0±0.0  (0.00) | 0.0 ± 0.0  (0.00) | 6.0 ± 3.1  (0.40) | 3.9 ± 2.5  (0.12) | ND |
|  | Ergosterol | 57.9 ± 8.8  (1.08) | 12.9 ± 3.9  (0.69) | 13.8 ± 2.7  (0.73) | 38.9 ± 1.2  (1.16) | 38.9 ± 12.6  (1.04) | 32.4 ± 23.7  (0.39) | 49.7 ± 18.9  (0.33) | 26.8 ± 3.0  (0.53) |

Legend ND indicates "not determined" or "below the limit of detection." An overview of the strains and inserts is provided in S1 Table. Sterols present at less than 1% were not included in the table.

**b**

**c**


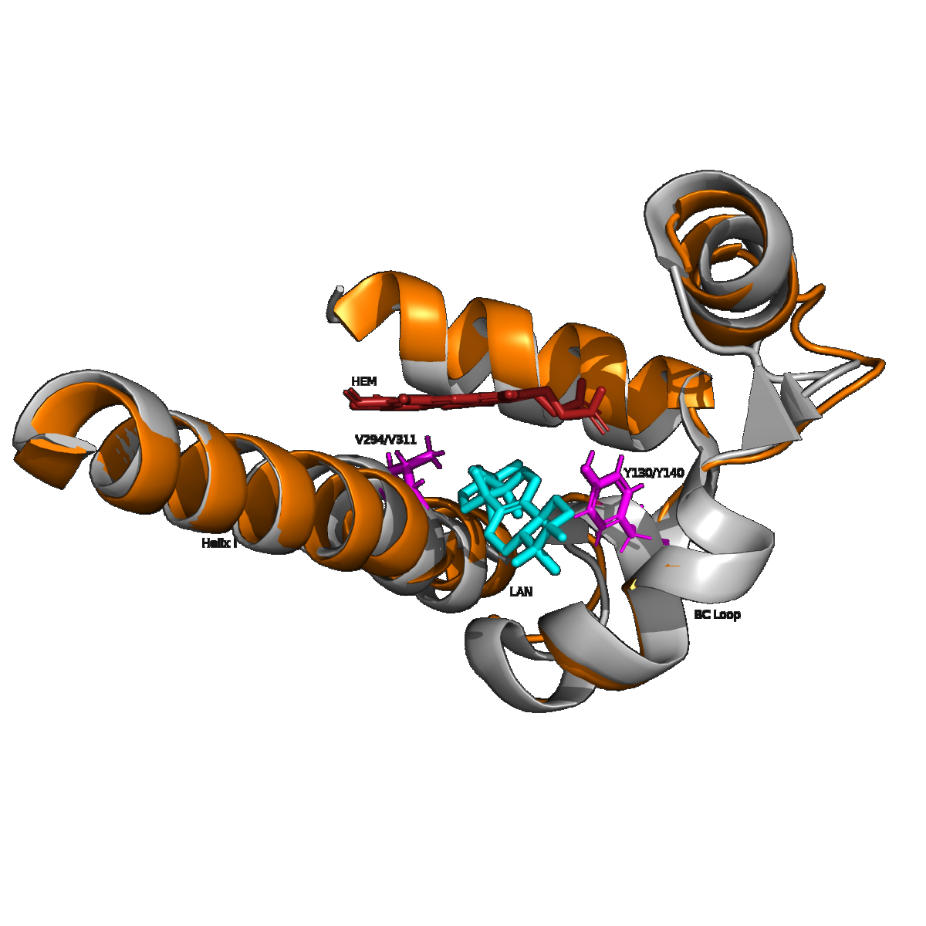


**a**

HEM

Helix I

BC Loop

LAN

V294/V311

Y130/Y140


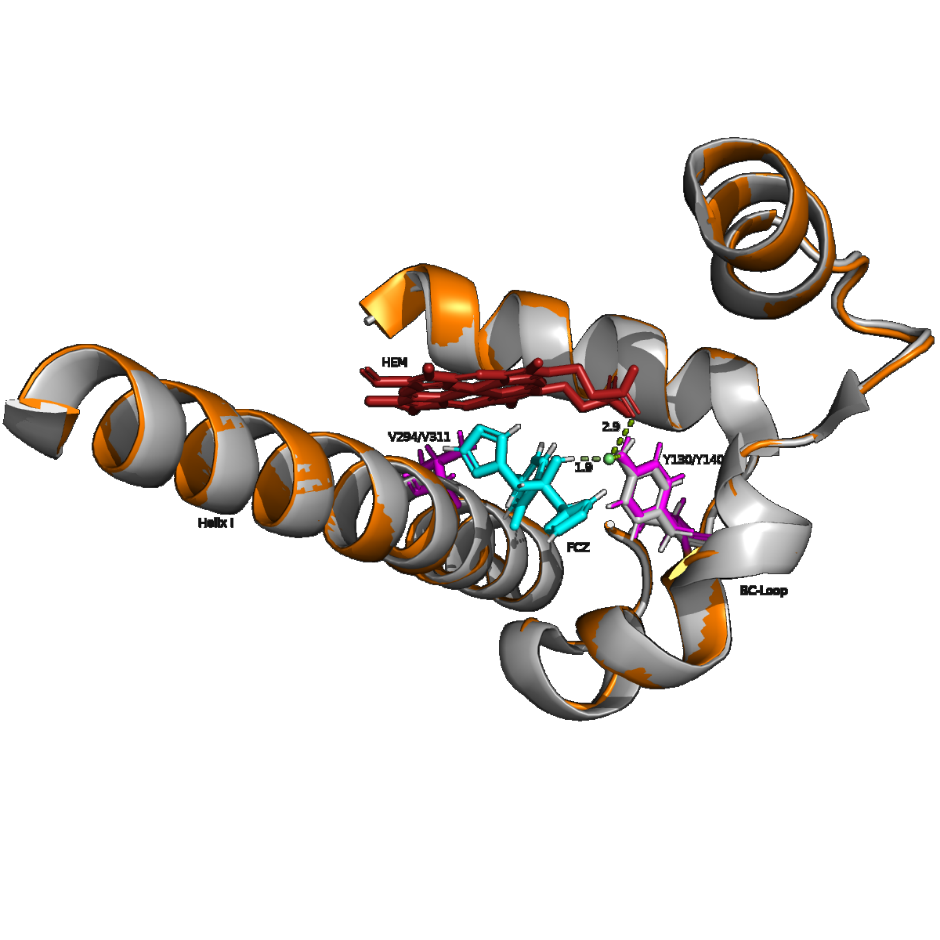


FCZ

Helix I

BC Loop

Y130/Y140

V294/V311

HEM

2.9

1.9


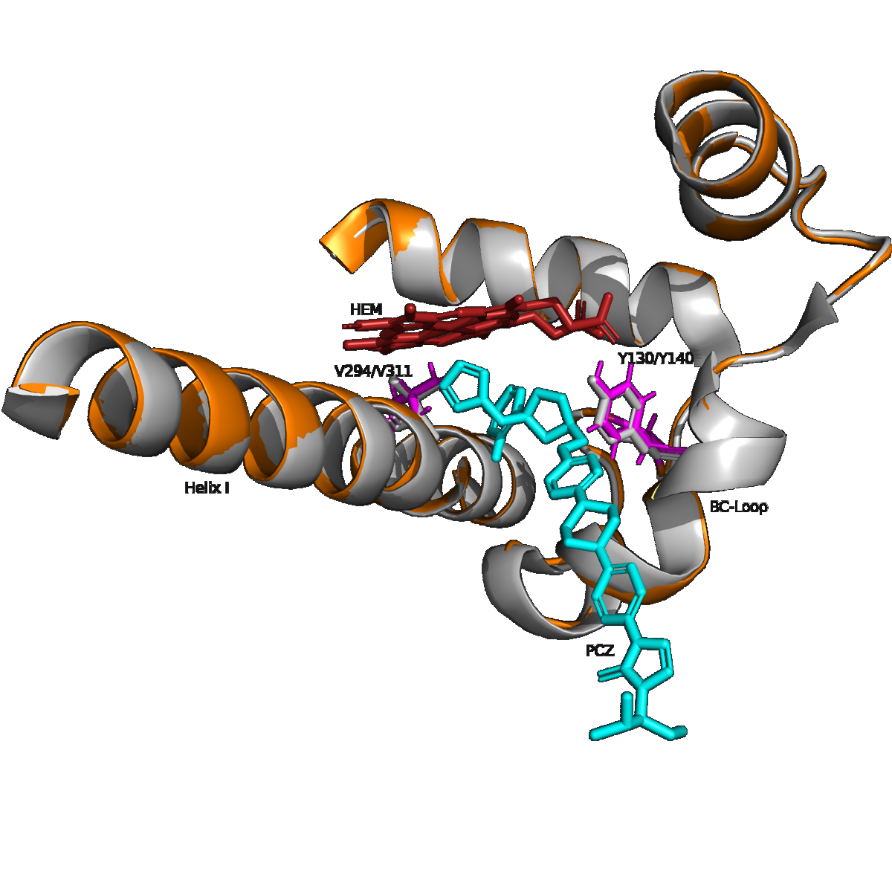


BC Loop

HEM

Helix I

PCZ

V294/V311

Y130/Y140

**Fig. S 12. In silico cartoon visualization of MluCyp51-F1 isoform with key ligands.** MluCyp51-F1 (orange) ligand binding pocket (Helix I and BC-loop) is aligned with crystal structures of ScErg11 (grey) incorporated with LAN (a, from PDB: 6UEZ), FCZ (b, from PDB: 4WMZ), and PCZ (c, from PBD: 6E8Q). Amino acid positions ScErg11 Y140, ScErg11 V311, MluCyp51-F1-Y130 and MluCyp51-F1 V294 are displayed as magenta-colored sticks. Green dashes indicate polar contacts to a water molecule (green dot).

Legend: HEM-heme, LAN-lanosterol, FCZ-fluconazole, PCZ-posaconazole


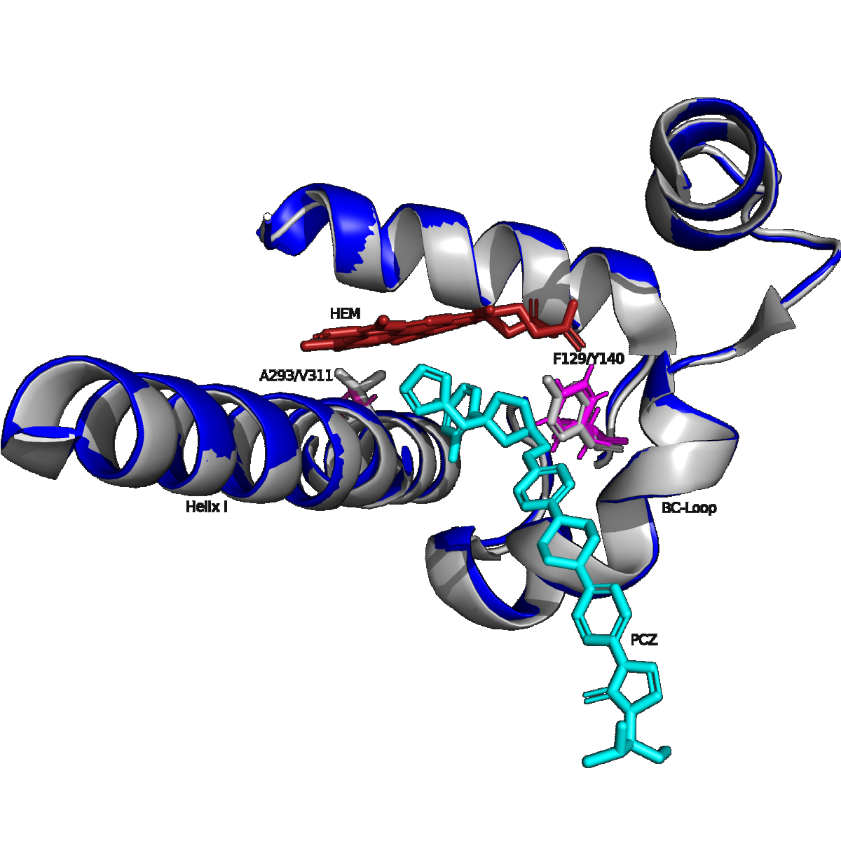

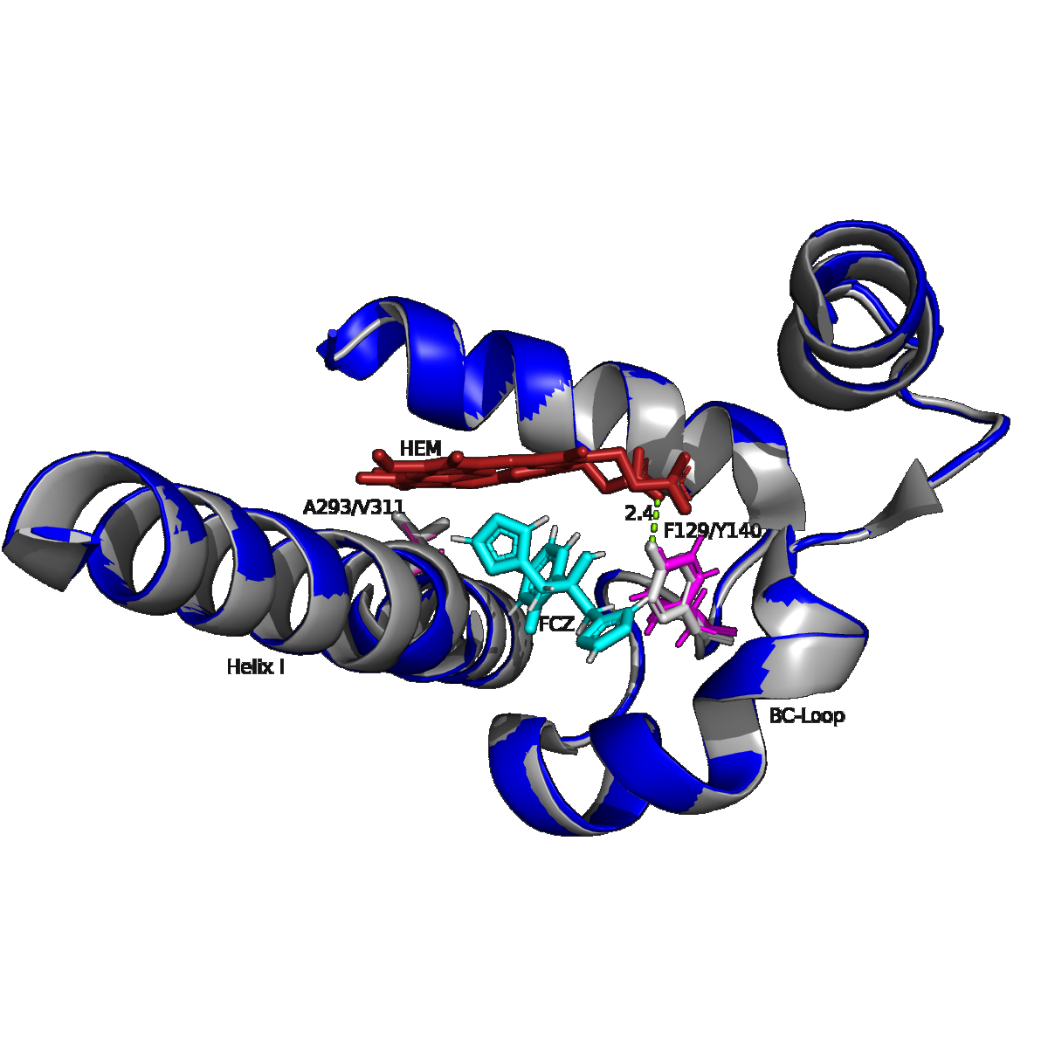


**a**

**c**

**b**


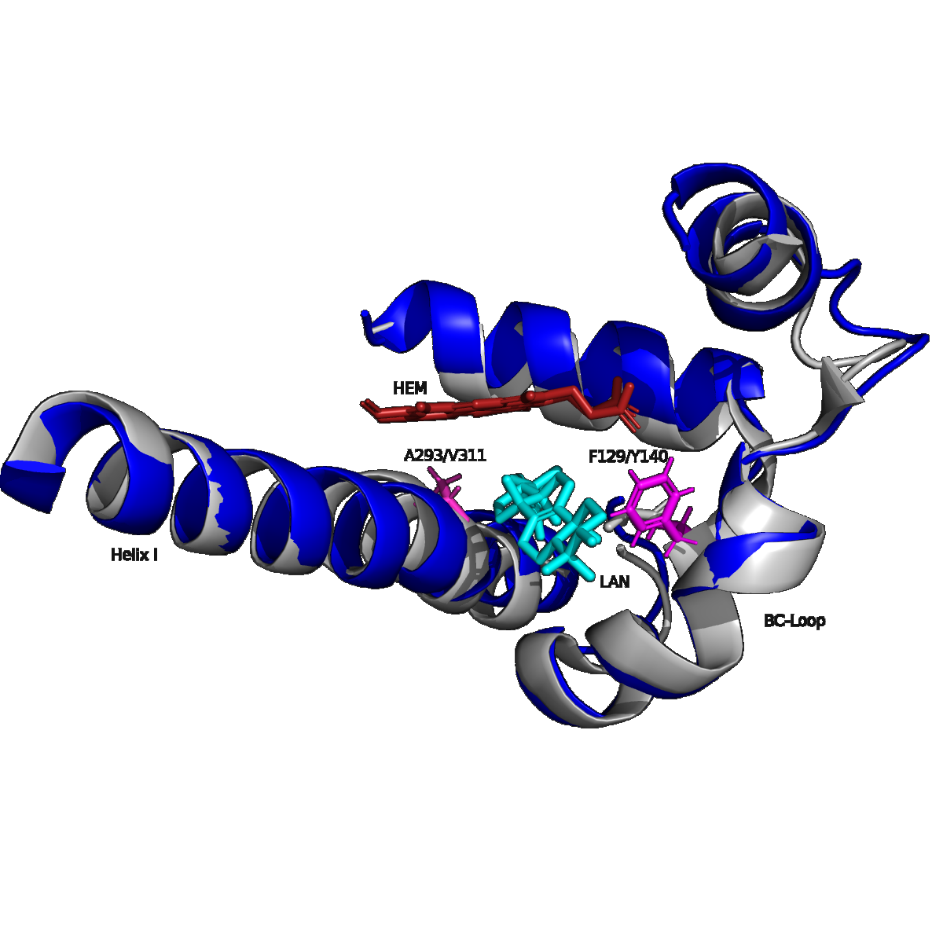


Helix I

BC Loop

A293/V311

F129/Y140

FCZ

2.4

**Fig. S 13. In silico cartoon visualization of MluCyp51- F5 isoform with key ligands.**

MluCyp51-F5 (blue) ligand binding pocket (Helix I and BC-loop) is aligned with crystal structures of ScErg11 (grey) incorporated with LAN (a, from PDB: 6UEZ), FCZ (b, from PDB: 4WMZ), and PCZ (c, from PBD: 6E8Q). Amino acid positions ScY140, ScV311, MluCyp51-F5 F129, and MluCyp51-F5 A293 are displayed as magenta-colored sticks. Green dashes indicate polar contacts and distances in Angstroms. Legend: HEM-heme, LAN-lanosterol, FCZ-fluconazole, PCZ-posaconazole.
